# Effectiveness of mHealth Interventions in the Control of Lifestyle and Cardiovascular Risk Factors in Patients After a Coronary Event: Systematic Review and Meta-analysis

**DOI:** 10.2196/39593

**Published:** 2022-12-02

**Authors:** Celia Cruz-Cobo, María Ángeles Bernal-Jiménez, Rafael Vázquez-García, María José Santi-Cano

**Affiliations:** 1 Faculty of Nursing and Physiotherapy University of Cádiz Cádiz Spain; 2 Institute of Biomedical Research and Innovation of Cádiz (INiBICA) Cádiz Spain; 3 Research Group on Nutrition, Molecular, Pathophysiological and Social Issues University of Cádiz Cádiz Spain; 4 Cardiology Unit Puerta del Mar University Hospital Cádiz Spain

**Keywords:** coronary disease, acute coronary syndrome, mobile health, smartphone, mobile apps, mobile phone

## Abstract

**Background:**

Coronary artery disease is the main cause of death and loss of disability-adjusted life years worldwide. Information and communication technology has become an important part of health care systems, including the innovative cardiac rehabilitation services through mobile phone and mobile health (mHealth) interventions.

**Objective:**

In this study, we aimed to determine the effectiveness of different kinds of mHealth programs in changing lifestyle behavior, promoting adherence to treatment, and controlling modifiable cardiovascular risk factors and psychosocial outcomes in patients who have experienced a coronary event.

**Methods:**

A systematic review of the literature was performed following PRISMA (Preferred Reporting Items for Systematic Reviews and Meta-Analyses) guidelines. A thorough search of the following biomedical databases was conducted: PubMed, Embase, Web of Science, SciELO, CINAHL, Scopus, The Clinical Trial, and Cochrane. Articles that were randomized clinical trials that involved an intervention consisting of an mHealth program using a mobile app in patients after a coronary event were included. The articles analyzed some of the following variables as outcome variables: changes in lifestyle behavior, cardiovascular risk factors, and anthropometric and psychosocial variables. A meta-analysis of the variables studied was performed with the Cochrane tool. The risk of bias was assessed using the Cochrane Collaboration tool; the quality of the evidence was assessed using the Grading of Recommendations, Assessment, Development, and Evaluation tool; and heterogeneity was measured using the *I*^2^ test.

**Results:**

A total of 23 articles were included in the review, and 20 (87%) were included in the meta-analysis, with a total sample size of 4535 patients. Exercise capacity measured using the 6-minute walk test (mean difference=21.64, 95% CI 12.72-30.55; *P*<.001), physical activity (standardized mean difference [SMD]=0.42, 95% CI 0.04-0.81; *P*=.03), and adherence to treatment (risk difference=0.19, 95% CI 0.11-0.28; *P*<.001) were significantly superior in the mHealth group. Furthermore, both the physical and mental dimensions of quality of life were better in the mHealth group (SMD=0.26, 95% CI 0.09-0.44; *P*=.004 and SMD=0.27, 95% CI 0.06-0.47; *P*=.01, respectively). In addition, hospital readmissions for all causes and cardiovascular causes were statistically higher in the control group than in the mHealth group (SMD=–0.03, 95% CI –0.05 to –0.00; *P*=.04 vs SMD=–0.04, 95% CI –0.07 to –0.00; *P*=.05).

**Conclusions:**

mHealth technology has a positive effect on patients who have experienced a coronary event in terms of their exercise capacity, physical activity, adherence to medication, and physical and mental quality of life, as well as readmissions for all causes and cardiovascular causes.

**Trial Registration:**

PROSPERO (International Prospective Register of Systematic Reviews) CRD42022299931; https://www.crd.york.ac.uk/prospero/display_record.php?RecordID=299931

## Introduction

### Background

Cardiovascular diseases (CVDs) are the main cause of death worldwide [1] according to data from the World Health Organization. They are considered to be responsible for 17.5 million deaths every year, which is 30% of those recorded worldwide [1]. In high-income countries, approximately 70% of CVD cases are attributed to modifiable risk factors, the most common being metabolic risk factors (obesity and cholesterol) and tobacco use [2].

Among CVDs, coronary artery disease (CAD) is the main cause of death and loss of disability-adjusted life years worldwide [3]. Much of this burden falls on low-income and medium-income countries, representing nearly 7 million deaths and 129 million disability-adjusted life years per year [4].

The secondary prevention of CAD is considered essential at present [5], as it has contributed significantly to the decrease in morbidity and mortality by facilitating the adoption of and adherence to healthy behavior, promoting an active lifestyle, and increasing adherence to drug treatment [5,6].

Thanks to the advances in medicine and technology, hospital stays after myocardial infarction have been shortened in recent years, meaning that health care professionals have fewer opportunities to inform patients about their disease during their admission [7].

Information and communication technology is becoming an increasingly important part of health care systems, including the innovative cardiac rehabilitation (CR) services through mobile phone and mobile health (mHealth) interventions [8]. mHealth technology can provide evidence-based guidance in an attractive, and user-friendly format, thus decreasing health care costs [9]. A meta-analysis [10] of 30 randomized trials including 7283 patients with CAD concluded that secondary prevention with telehealth programs can be used instead of, or together with, traditional CR and is associated with greater control of cardiovascular risk factors and fewer clinical events. This study, however, used different kinds of telehealth interventions in each trial (internet, telephone calls, SMS text messages, and mobile apps).

Early secondary preventive care patients recently discharged after acute coronary syndrome was shown to promote adherence to drug treatment and facilitate the control of changes in cardiovascular risk factors. However, because of the COVID-19 pandemic, it is likely that the uptake and availability of secondary prevention strategies have been affected, as CR programs may have been suspended or patients avoided or could not go to health centers [11,12]. Therefore, innovative secondary prevention and CR strategies are needed to be implemented to increase long-term adherence to a healthy lifestyle.

### Objectives

Despite the exponential growth in and availability of smartphone technology to provide a new tool to optimize the secondary prevention of heart diseases, no systematic reviews have been published that focus exclusively on the effectiveness of mHealth involving mobile apps as a way of providing digital health and its secondary prevention components to patients who have experienced a coronary event. Thus, the aim of this review was to determine the effectiveness of the different means of providing mHealth programs in changing lifestyle behavior, promoting adherence to treatment, and controlling modifiable cardiovascular risk factors and psychosocial outcomes.

## Methods

### Search Strategy

A systematic review of the literature was performed following the PRISMA (Preferred Reporting Items for Systematic Reviews and Meta-Analyses) guidelines [13]. A thorough search was conducted of the following biomedical databases between June and November 2021: PubMed, Web of Science, Scopus, SciELO, CINAHL, Cochrane, and The Clinical Trial. Manual searches of the references from other reviews and meta-analyses were also performed to find more studies. The search terms included the following: *coronary syndrome, infarction, acute coronary syndrome, coronary disease, mHealth, mobile applications,* and *smartphone*, which were combined with each other using the Boolean operators (AND/NOT) and the appearance of these terms into Title or Abstract (Multimedia Appendix 1). Truncation (*) was applied when necessary to improve the search results. The search was limited to the time frame from 2015 to 2021. The search protocol was registered with PROSPERO (International Prospective Register of Systematic Reviews; registration number: CRD42022299931).

### Inclusion and Exclusion Criteria: Selection of Studies

Studies were included if they complied with the inclusion criteria, namely randomized controlled trials (RCTs) in which an intervention had been performed consisting of a telehealth or mHealth program by means of a mobile app in patients with coronary heart disease and included the following outcome variables: change in lifestyle behavior (diet, physical exercise, and treatment adherence) and control of cardiovascular risk factors (tobacco, blood sugar, systolic blood pressure [SBP], diastolic blood pressure [DBP], total cholesterol, low-density lipoprotein [LDL] cholesterol, and high-density lipoprotein [HDL] cholesterol); anthropometric variables (waist circumference and BMI); and psychosocial variables (anxiety, depression, and stress).

Studies that used SMS text messages without an app or web portal and included participants who had experienced a stroke or another CVD were excluded.

### Study Selection

Two researchers independently examined the identified articles using the search strategy described in the *Search Strategy* section. First, the titles and abstracts of the articles were checked, and 58 articles were selected for the whole text to be read. A critical reading was performed, and a decision was made regarding whether the articles complied with the inclusion criteria. If there was any discrepancy regarding which articles were eligible for selection, a third reviewer intervened to resolve the problem, helping to reach a final agreement. The quality of the included RCTs was assessed using the Grading of Recommendations, Assessment, Development, and Evaluation tool [14]. This tool provides an approach to grading the quality or certainty of evidence and strength of recommendations. It is a framework for evaluating the effectiveness of systematic reviews. The Grading of Recommendations, Assessment, Development, and Evaluation tool speciﬁes 4 categories for the quality of a body of evidence: high, moderate, low, and very low (Multimedia Appendix 2). The risk of bias was assessed using the Cochrane tool [15], which is used to assess the methodology of scientific evidence in systematic reviews for the individual analysis of included RCTs, addressing 7 specific domains: random sequence generation, allocation concealment, blinding of participants and personnel, blinding of outcome assessment, incomplete outcome data, selective reporting, and other bias. Publication bias was assessed using funnel plots (Multimedia Appendix 3).

### Data Extraction Synthesis and Analysis

A total of 23 studies were included in the systematic review, of which 20 (87%) were included in the meta-analysis. The Cochrane Review Manager (RevMan 5.4; The Cochrane Collaboration) software was used for the statistical analysis. Differences in the effects of mHealth interventions and standard health care were examined by means of the inverse variance method. The difference of means was used as the statistic to analyze the effect, and the standardized difference of means was used when variables with different measurement scales were compared, and a 95% CI was given for each effect size. Risk difference was assessed for the qualitative variables. To test the hypothesis, the *P* value was set at <.05 with 2 tails. The analysis was performed in general using the random-effects model, and when heterogeneity was 0%, the fixed-effects model was used. Heterogeneity was assessed by means of the *I*^2^ statistic, which is a useful statistic for quantifying inconsistency. It describes the percentage of the variability in effect estimates that is because of heterogeneity rather than sampling error. A value of *I*^2^<25% was considered low heterogeneity, *I*^2^ from 25% to 50% moderate heterogeneity, and *I*^2^>50% high heterogeneity [16]. The sensitivity of the meta-analysis was tested [16]. Forest plots were constructed to visualize the results.

## Results

### Selection of Studies

**Figure 1:**
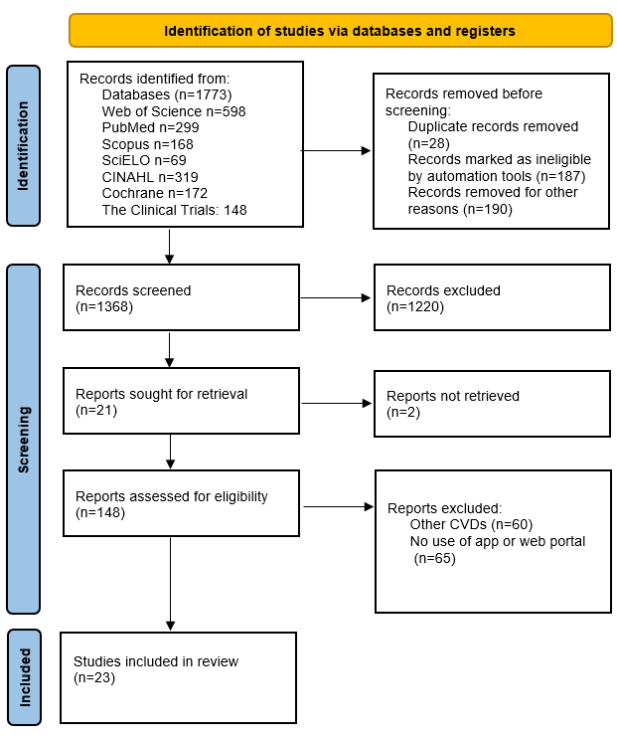
The PRISMA (Preferred Reporting Items for Systematic Reviews and Meta-Analyses) flow diagram of the study selection process. CVD: cardiovascular disease.

The search provided a total of 1773 articles that were distributed among the following databases: Web of Science (n=598, 33.73%); PubMed (n=299, 16.86%); Scopus (n=168, 9.48%); SciELO (n=69, 3.89%); CINAHL (n=319, 17.99%); Cochrane (n=172, 9.7%); and The Clinical Trial (n=148, 8.35%). A total of 23.18% (411/1773) of articles were identified as duplicates and hence removed. First, the titles and then the abstracts were checked using the inclusion and exclusion criteria. Eventually, 8.35% (148/1773) of articles were selected for the whole text to be read, of which 15.5% (23/148) were chosen for the review and 13.5% (20/148) for the meta-analysis. Figure 1 shows a summary of the selection of studies using the PRISMA flow diagram.

### Characteristics of the Studies

All the articles that were selected to be part of the review were RCTs, with a total of 4535 patients. Among the 24 variables analyzed in the RCTs, 54% (13/24) presented evidence of high or moderate quality and 46% (11/24) of variables provided low or very low quality of evidence. The follow-up duration of the intervention ranged from 2 to 26 months, with the most frequent duration being 6 months. The age of the patients in these clinical trials ranged from 55 to 66 years and 81.32% (3688/4535) of the patients were male.

In the included studies, the control group received “usual health care” or “standard medical care” after the coronary event. In general, the interventions were conducted by a multidisciplinary team of nurses, cardiologists, physiotherapists, nutritionists, specialists in sports medicine, and exercise physiologists.

The patient dropout rate across the studies did not exceed 20%, except in the trial by Skobel et al [17], which had a dropout rate of 65.5% in the intervention group and 34% in the control group. The main reasons for participants dropping out were the health care professionals being unable to contact the participants, the participants wishing to withdraw from the study, and health problems making it impossible for them to continue.

The most commonly studied variables were SBP, DBP, and lifestyle, whereas the least frequently analyzed were c-reactive protein, which was studied only by 2 authors [13,14]; improvement in diet, studied by Choi et al [18] using the “Mediterranean diet score” and Widmer et al [19] using the “food score”; and nicotine dependence (by means of the Fagerström Test), analyzed only in the RCT by Fang et al [20]. An economic assessment analyzing the profitability of the intervention was conducted only by Frederix et al [21] and Maddison et al [22].

Multimedia Appendix 4 [17-39] shows a summary of the design of the included studies, the components of the mHealth systems used, and the initial characteristics of the patients included.

### Risk of Bias in the Included Studies

**Figure 2:**
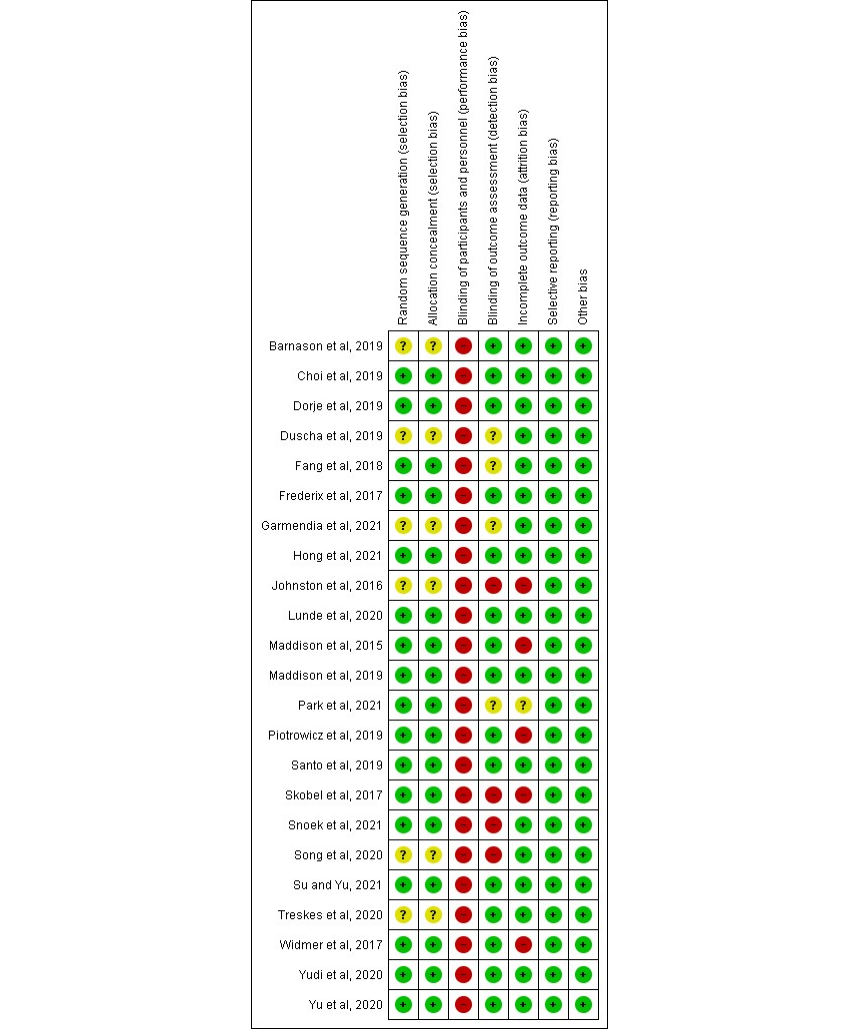
Risk of bias summary: authors’ judgments about each risk of bias item for each included trial.

**Figure 3:**
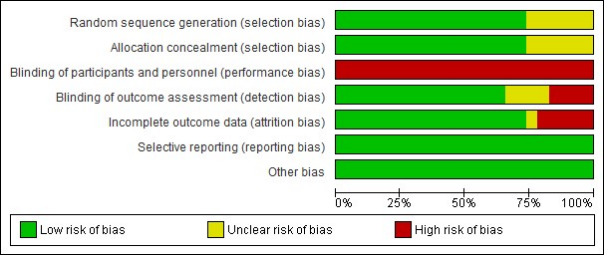
Risk of bias graph: authors’ judgments about each risk of bias item presented as percentages across all included trials.

The risk of bias in the RCTs included in this review is summarized in Figure 2 [17-39] and Figure 3. The random sequence generator and the concealment of the allocation of the patients recruited to the RCT were accurately presented in most of the studies, and they had been classified as low risk. The methods used for the randomization were a computer-generated random sequence, 2-tailed *t* test, and permuted block technique. In total, 26% (6/23) of studies did not include information about the allocation concealment method used, so they were classified as presenting an unclear risk of bias because of the lack of specific information [34-39].

Owing to the nature of these RCTs (N=23), the participants, and sometimes the medical professionals, could not be blinded; therefore, all the trials were considered to present a high risk of concealment bias. The researcher assessing the results was not blinded in 17% (4/23) of studies [17,18,29,37]; in 17% (4/23) of other studies, the concealment bias was not clear, so they were categorized as having unclear risk [20,26,35,39], and in the 22% (5/23) of the remaining studies, specific details of the blinding of the assessors were given.

Regarding attrition bias, 22% (5/23) of the trials were considered high risk because of incomplete results data [19,27,36,40] and a dropout rate of >20% [17]. The study by Park et al [26] was classified as having an unclear risk of bias, as it was a pilot RCT reporting preliminary results. In contrast, 74% (17/23) of studies were considered to present a low risk of attrition bias as they provided clear and detailed descriptions, there were no missing results data, and the percentage of dropouts was <20%.

All the trials included in the review were classified as having a low risk of reporting bias because of the following reasons: the trials had study protocols that were readily available; the results studied were previously specified; or if the study protocol was not available, it was clear that all the expected results were included.

Finally, regarding other possible risks of bias, all the studies were classified as low risk, as the patients who participated in the trials provided their written, informed consent to participate in the study. All the RCTs were approved by the ethics committee of the institution where the trial was conducted, and their ethics approval statements were included in the texts.

In summary, most of the trials were assessed as having moderate risk, as it was not possible to blind all the participants because of the nature of these RCTs.

### Effects of the Interventions on the Results

#### Blood Lipids

A total of 39% (9/23) of the trials provided data on the plasma concentrations of total cholesterol and LDLs from a total of 1211 participants. Santo et al [28] and Snoek et al [29] did not provide information about HDL cholesterol despite reporting data on LDL cholesterol, and the sample for analyzing HDL cholesterol included 943 patients. Triglycerides were evaluated in 26% (6/23) of studies, with a total sample size of 889 patients. High heterogeneity was found in the studies analyzing total and LDL cholesterol levels.

The meta-analysis of the included trials did not show significant differences in total cholesterol (*P*=.44), LDL cholesterol (*P*=.35), HDL cholesterol (*P*=.21), and triglycerides (*P*=.72), although favorable outcomes were found in the mHealth groups (Multimedia Appendix 5 [17,18,21,25,27-29,31,32]).

#### Blood Pressure

A total of 57% (13/23) of studies with high heterogeneity (*I*^2^=78%) reported the SBP of 2459 included patients, and 52% (12/23) of studies, which also had high heterogeneity (*I*^2^=66%), informed about the DBP of 2187 patients. Dorje et al [23] did not provide data about DBP, although data about SBP after the intervention were included. No differences were found in either SBP (*P*=.99) or DBP (*P*=.36) between the groups after the interventions (Multimedia Appendix 6 [17,18,21,25,27-29,31,32,34]).

#### Body Composition

A total of 39% (9/23) of trials with high heterogeneity (*I*^2^=88%) studied the BMI (*P*=.97) of a total of 1986 patients. After the mHealth interventions, no significant differences in BMI were found between the groups. Neither was there a significant difference in waist circumference measurements between the 2 groups. This measurement was analyzed by 3 studies with high heterogeneity (*I*^2^=56%) with a sample of 376 patients (Multimedia Appendix 6).

#### Glycated Hemoglobin and Basal Blood Glucose

A total of 13% (3/23) of studies with high heterogeneity (*I*^2^=76%) evaluated glycated hemoglobin levels in a sample of 382 participants. Although the decrease was greater in the mHealth group, the difference was not statistically significant (glycated hemoglobin, *P*=.23 and basal blood glucose, *P*=.54). Fasting blood sugar levels were also reported in 13% (3/23) of homogeneous trials (*I*^2^=0%), with no significant improvements being found (Multimedia Appendix 7 [17,18,21,25,27-29,31,32,34,36]).

#### Heart Rate

Heart rate (*P*=.10) was lower in the mHealth groups, but the differences were not significant. A total of 13% (3/23) of studies with high heterogeneity (*I*^2^=65%) evaluated this value in a sample of 494 patients (Multimedia Appendix 7).

#### Exercise Capacity

**Figure 4:**
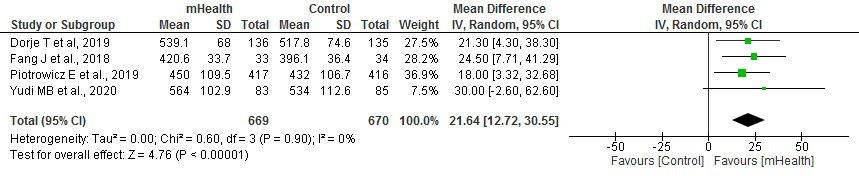
Forest plots for changes in the 6-minute walk test. IV: instrumental variable; mHealth: mobile health.

A total of 17% (4/23) of homogeneous studies (*I*^2^=0%) analyzed exercise capacity by means of the 6-minute walk test (6-MWT) with a sample of 1339 patients. The results of the meta-analysis showed that exercise capacity as measured by this test was significantly higher in the mHealth groups (*P*<.001; Figure 4 [20,23,27,31]).

Another outcome measure of exercise capacity was the peak oxygen consumption, studied in 8 trials with high heterogeneity (*I*^2^=64%) with a sample of 1512 patients, although the results were not significant (Multimedia Appendix 8 [17,21,25,27,29,32,35,37]).

#### Physical Exercise

**Figure 5:**
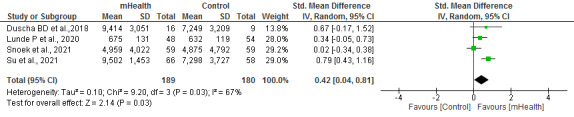
Forest plots for changes in physical exercise. IV: instrumental variable; mHealth: mobile health.

A total of 17% (4/23) of studies with high heterogeneity (*I*^2^=67%) analyzed physical exercise (steps/day, time until exhaustion, or the International Physical Activity Questionnaires questionnaire). The meta-analysis of the included trials showed a significant improvement in physical activity among the participants in the mHealth groups compared with those receiving standard health care (*P*=.03; Figure 5 [29,32,33,35]).

#### General Quality of Life

Health-related quality of life was studied in 39% (9/23) of RCTs with moderate heterogeneity (*I*^2^=47%) with a sample of 1741 patients, using the following validated questionnaires: European Quality of Life-5 Dimension (visual analog scale and index), Partners in Health scale, 36-Item Short Form Health Survey, Quality of Life after Myocardial Infarction questionnaire, and MacNew Heart Disease Health-Related Quality of Life questionnaire. The scores on these questionnaires were higher in the mHealth groups, but the differences did not reach statistical significance (Multimedia Appendix 9 [17,20,23-25,27,29,31,32,36]).

#### Physical and Mental Dimensions of Quality of Life

The physical and mental dimensions of quality of life were analyzed in 22% (5/23) of studies, with a sample of 620 patients. The following validated questionnaires were used in these trials: 12-Item Short Form Health Survey, 36-Item Short Form Health Survey, Health-Related Quality of Life, and World Health Organization Quality of Life: Brief Version.

**Figure 6:**
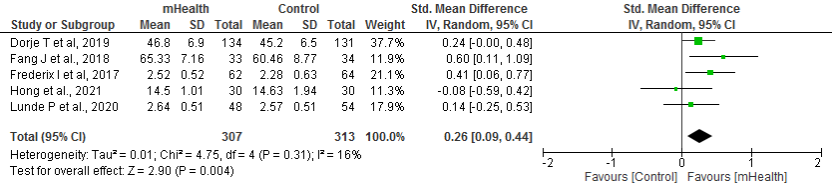
Forest plots for changes in quality of life (physical dimension or physical health). IV: instrumental variable; mHealth: mobile health.

**Figure 7:**
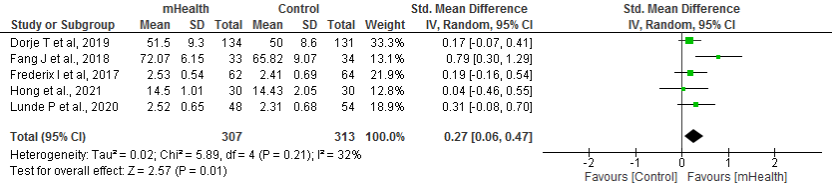
Forest plots for changes in quality of life (mental dimension or mental health). IV: instrumental variable; mHealth: mobile health.

In both the physical (*I*^2^=16%) and mental (*I*^2^=32%) dimensions, significantly higher scores were obtained in the groups that received the mHealth intervention than in the control group (*P*=.004 and *P*=.01, respectively; Figures 6 and 7 [20,21,23,24,32]).

#### Anxiety and Depression

Depression was analyzed in 22% (5/23) of trials with moderate heterogeneity (*I*^2^=40%), and anxiety was analyzed in 17% (4/23) of homogeneous studies (*I*^2^=0%). Fang et al [20] did not report data on depression, despite reporting data on anxiety. Anxiety was measured using the validated questionnaires, Generalized Anxiety Disorder-7 and Hospital Anxiety and Depression Scale- Anxiety. In a sample of 612 patients, no significant difference was found between the anxiety scores in both groups. Nor were there significant differences in the depression scores of a sample of 679 patients (Multimedia Appendix 9; anxiety, *P*=.30 and depression, *P*=.84). The questionnaires used were Patient Health Questionnaire-9, Hospital Anxiety and Depression Scale-Depression, and Calgary Depression Scale.

#### Adherence to Medication

**Figure 8:**
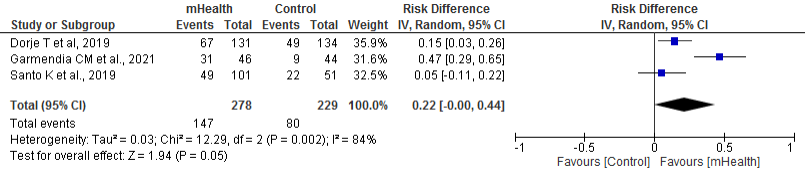
Forest plots for changes in adherence to medication. IV: instrumental variable; mHealth: mobile health.

Three authors studied adherence to medication using the 8-item Morisky Medication Adherence Scale and a self-reported questionnaire in a survey of 507 patients (*I*^2^=84%). Adherence to medication was greater in the mHealth group (*P*=.05; Figure 8 [23,28,39]).

#### Mortality

A total of 13% (3/23) of studies analyzed the difference in mortality between the groups. In the meta-analysis, with a sample of 2010 patients, no significant differences in all-cause mortality (*P*=.64) were found (Multimedia Appendix 10 [27,30,38]).

#### Rehospitalization

**Figure 9:**
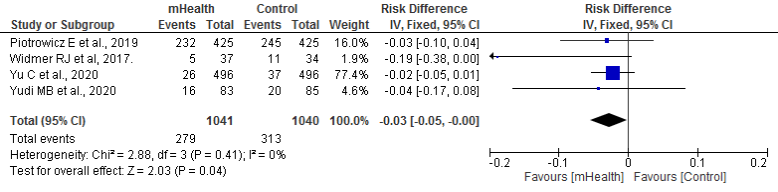
Forest plots for changes in rehospitalizations for all causes. IV: instrumental variable; mHealth: mobile health.

**Figure 10:**
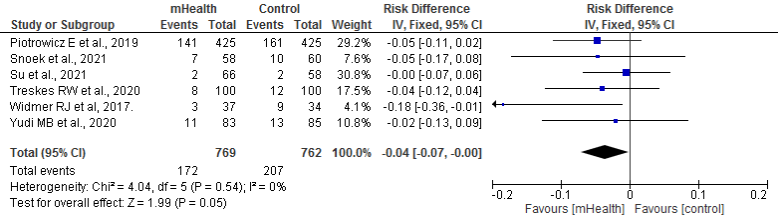
Forest plots for changes in rehospitalizations for cardiovascular causes. IV: instrumental variable; mHealth: mobile health.

Regarding the rehospitalizations of patients during the study period in each RCT, the meta-analysis showed that rehospitalizations for both all causes (*P*=.04) and cardiovascular causes (*P*=.05) were statistically higher in the control group than in the mHealth group. These studies were homogeneous (*I*^2^=0%; Figure 9 [19,27,30,31] and Figure 10 [19,27,29,31,33,38]).

Furthermore, a sensitivity analysis was performed by excluding each study sequentially to determine the influence of any single study on the robustness of the results, revealing no substantial difference in the overall effect for the 6-MWT, quality of life, physical activity, and rehospitalizations (Multimedia Appendix 11 [19-21,23,24,27,29-33,35,38]).

## Discussion

### Principal Findings

This study presents an assessment of evidence from RCTs that compared the effects of mHealth and standard interventions on lifestyle, adherence to treatment, and changes in cardiovascular risk factors after a coronary event. This meta-analysis provides evidence of the favorable effects of mHealth interventions on the variables analyzed. The fact that the studies chosen for this review were published in recent years is proof of the growing interest in mHealth interventions as a resource aimed at improving the secondary prevention of CAD.

Keeping blood lipids and blood pressure under control are very important objectives in the secondary prevention of CVDs. A meta-analysis conducted by Gencer et al [41] involving 21,492 patients aged >75 years reported that a 1 mmol/L decrease in LDL cholesterol significantly reduced the risk of vascular events by 26%. Regarding blood pressure, a meta-analysis by Ettehad et al [42] concluded that a decrease of 10 mmHg in SBP reduces the risk of important cardiovascular events by approximately 20%, CAD by 17%, and all-cause mortality by 13%. Regarding lipid variables and blood pressure, our meta-analysis did not reveal a significant advantage of smartphone technology compared with standard health care after a coronary event, possibly owing to the fact that in these cases, intensive drug treatment is prescribed that has a similar effect on patients participating in an mHealth program to those receiving standard health care. However, the results could also be a result of the high heterogeneity between the studies that measured the total and LDL cholesterol levels. Regarding SBP and DBP, our results agree with those obtained in recent meta-analyses [43-45]. However, they do not coincide with other meta-analyses that obtained improvements in DBP only [46]. They also do not coincide with the meta-analysis published by Kavradim et al [47], who observed improvements in both SBP and DBP. Concerning lipid variables, our results are in line with those published by Huang et al [44] and Al-Arkee et al [45]. In contrast, Akinosun et al [43] found improvements in LDL, HDL, and total cholesterol levels but not in triglycerides. Xu et al [46] observed significant improvements only in HDL and total cholesterol, but not in LDL cholesterol, and Kavradim et al [47] found significant improvements in total cholesterol and triglycerides, but not in LDL and HDL cholesterol. The digital technology interventions analyzed were not based on the use of mobile phone apps, but rather on SMS text messages and web-based coaching.

The increased prevalence of obesity has become an important public health concern worldwide. Total and abdominal adiposity during adolescence is associated with atherosclerosis in adulthood and insulin resistance [48]. Abdominal obesity is the most frequently observed component of metabolic syndrome (the cluster of abdominal obesity, dyslipidemia, hyperglycemia, and hypertension). The mean prevalence of metabolic syndrome among 24,670 participants aged 35-74 years from 10 autonomous communities in Spain was found to be 31% and is associated with a 2-fold increase in the risk of CAD and a 1.5-fold increase in the risk of all-cause mortality [49]. In our meta-analysis, mHealth interventions did not lead to a significant reduction in the patients’ BMI and waist circumference. In this sense, it is worth highlighting that only a few RCTs have measured waist circumference despite the positive correlation between abdominal obesity and atherosclerosis. This finding is in accordance with the results of Akinosum et al [43] and Huang et al [44] who also did not observe improvements in BMI; however, a recent meta-analysis [46] did find a reduction in BMI and waist circumference although few RCTs were included in the analysis. Moreover, each trial used a different kind of digital intervention (telephone calls, remote monitoring with smartphones, SMS text messages, medication reminder apps, conference call sessions, emails, or web apps).

A high blood glucose level is also an important risk factor leading to the onset and development of CAD. A recent meta-analysis concluded that prediabetes is associated with a greater risk of all-cause mortality and CVD in the general population and in patients with atherosclerotic CVD [50]. Our study, similar to the one performed by Akinosun et al [43], did not find a significant decrease in glycated hemoglobin or fasting blood glucose levels in the mHealth group. These results may be due to the fact that few RCTs included these variables and also because of the differences in the duration of the intervention and monitoring periods.

Regarding the number of people who had stopped smoking at the end of the intervention, the percentage was high in both groups (standard care and mHealth), but the results were not statistically significant. These findings are similar to those reported in the meta-analyses by Akinosun et al [43] and Huang et al [44], who did not report a significant difference in the prevalence of tobacco use between the groups at the end of the study. However, another meta-analysis did conclude that telehealth inventions have a statistically significant beneficial effect, albeit a small one, on stopping smoking in patients with CAD [47] using SMS text messages, telephone calls, and telemonitoring. Akinosun et al [43] observed that mHealth interventions appeared to be more effective in improving healthy behaviors than unhealthy ones (alcohol consumption and smoking). One reason for this could be that tobacco cessation interventions use behavioral change techniques, which include social support and group discussions, and such techniques are less frequently included in mHealth interventions.

Physical inactivity is independently associated with 12.2% of the global burden of acute myocardial infarction [51]. Consequently, physical activity is considered the cornerstone on which changes in lifestyle to prevent CVD must be based, and a dose-response relationship exists between 6-MWT and the risk of future cardiovascular events. Moreover, 6-MWT is a known predictor of cardiovascular events in patients with CAD, even after adjusting for cardiovascular risk factors [31]. Therefore, the results obtained in the meta-analysis are encouraging because the use of mHealth strategies is seen to result in favorable changes in exercise capacity, which can have a positive impact on the secondary prevention of future cardiovascular events. In addition, no meta-analysis published to date was found to have studied physical capacity with 6-MWT for mHealth interventions in patients who have experienced a coronary event. These results align with existing systematic reviews of mHealth in cardiovascular patients, which demonstrate improvements in physical activity with digital technology [43,47]. However, in the systematic review by Huang et al [44], they did not observe an increase in physical activity with mHealth interventions.

Many patients do not comply with lifestyle recommendations or do not take their medication as prescribed after a cardiovascular event. Adherence to treatment by patients who are prescribed cardiovascular medication is estimated to be approximately 51% a year after a myocardial infarction [52]. Among these patients, 30% interrupt their treatment 3 months after the first infarction, whereas 50% do so after 1 year [53,54]. The results of our meta-analysis show that mHealth interventions have a positive impact on adherence to medication although there is high heterogeneity among the studies and only a few include this variable. These results are in line with those found in a recent meta-analysis [45] assessing the effects of mobile phone health care apps on adherence to medication in patients with CVD, with the apps being based on medication reminders on the mobile device. Meta-analyses by Kavradim et al [47] and Akinosun et al [43] also found increased adherence to medication with telehealth interventions in the secondary prevention of CAD and patients with CVD, respectively.

CAD is one of the main causes of disability and loss of health-related quality of life among patients with this disease [4]. Thus, improving quality of life is one of the most important objectives to be achieved with these patients. Assessing quality of life allows for the subjective evaluation of an individual’s health and the determination of the impact of the disease and its treatment on their daily life. In our meta-analysis, the scores in both the physical and mental dimensions of quality of life were statistically higher in the mHealth group, a finding that may be related to the capacity of mHealth interventions to provide remote health care to patients and answer their questions at any time. In one of the meta-analyses [44], no significant differences were observed in the quality of life between telehealth interventions and CR in patients with CAD. In general, few meta-analyses include quality of life among the study variables, which makes it difficult to make comparisons [55].

Several studies have reported that anxiety and depression are also independent risk factors for cardiovascular morbidity and mortality [56,57]. Therefore, dealing with stress, psychosocial risk factors (eg, lack of social support), and other mood disorders is an objective that takes precedence [53]. In our meta-analysis, the levels of anxiety and depression in the mHealth group were not statistically different from those in the patients receiving standard care. Our results agree with the meta-analysis by Huang et al [44]. Nevertheless, the meta-analysis by Xu et al [46] reported that mHealth strategies could alleviate depression in patients with coronary cardiopathy, but it had no effect on anxiety. These results may also be due to the fact that few RCTs include and analyze psychosocial variables.

In our meta-analysis, we did not observe differences between the intervention and usual care groups in terms of mortality, but we did find a reduction in hospitalizations for all causes and for cardiovascular causes with the digital intervention. However, these variables were not included in other meta-analyses, which makes their comparison difficult.

This review found that although the usability, viability, and acceptance of mHealth tools for modifying cardiovascular risk factors and lifestyle were included as variables in a few studies, they were highly valued by the patients. A study by Johnston et al [36] using the System Usability Scale observed that 97.5% of the patients in the intervention group said at the end of the study that they would recommend the tool to other patients in the same situation. Moreover, 68.4% of the patients reported being willing to continue using the web-based tool, and >80% found that the patient support tool provided relevant information about the disease and increased their knowledge and motivation to follow a healthier lifestyle. Al-Arkee et al [45] also reported usability results that were favorable to the intervention.

The heterogeneity of some of the variables studied was high, possibly because of the small sample sizes; different monitoring durations of the RCTs; differences in the age of the participants; and different settings in which the mHealth programs took place (hospital, home, or outpatient clinics).

In general, systematic reviews include interventions with different technologies such as mobile phones, websites, and software apps, but they do not usually compare these technologies with each other. However, the meta-analysis by Xu et al [46] conducted subgroup analyses to compare simple (telephone calls, messages, and WeChat messages) and complex (self-developed apps, wearable devices, medical platforms, and videoconferencing) mHealth interventions. The results of the subgroup analysis showed that the simple mHealth group was more conducive to controlling risk factors than the complex mHealth group. These results may be related to the age of the patients. CVD occurs frequently in middle-age people and older adults who are from a different technological generation, and they find it difficult to use technological devices. The complicated interface design with a small font size of some complex mHealth interventions or the handling difficulties of wearable devices may reduce the engagement of patients with CVD.

Cell phones are considered efficient digital devices and have been the most widely studied because of their affordability and ease of use; however, smartphones may have advantages because of additional interaction features.

### Limitations

Regarding the limitations of our study, it is worth mentioning aspects such as the fact that the participants in the included RCTs enrolled voluntarily by signing an informed consent form, which probably introduced a selection bias, as these patients might have been more motivated to adhere to secondary prevention than others who chose not to participate. Another limitation could be that to participate in the mHealth programs, the patients had to have a mobile phone or a tablet with an internet connection, which could suggest that the participants were younger. However, this limitation seems to be of little importance because nowadays, >75% of the world population has a mobile telephone with internet access and >57% of homes have an internet connection. In Europe, these figures are even higher, reaching 99% and 86%, respectively [58].

Another consideration is that the trials used nonvalidated self-reported questionnaires to analyze some objectives, resulting in the generalizability and coherence of the studies being variable. More studies are required to examine the long-term impact of smartphone-based interventions on people who have experienced a coronary event with regard to heart-related mortality and hospital admissions, as these are important measures of the success of secondary prevention strategies.

### Strengths

A strength of our meta-analysis is that RCTs with very similar interventions were selected, involving the use of an app or web portal and programs based on SMS text messages or reminders and telephone calls were excluded. As a result, the interventions analyzed used the newest and most up-to-date technology. To the best of our knowledge, this is the first meta-analysis to group these interventions based on mobile apps for secondary prevention exclusively in patients with CAD after a coronary event, not with risk of CVD. Another strength is the inclusion of many kinds of behavioral, metabolic, and psychosocial variables, providing a broad view of the results being obtained with mHealth technology. All the studies included in this review and meta-analysis were RCTs that are the key to scientific evidence in clinical research. In addition, these clinical trials were conducted in a wide variety of countries in Europe, America, Asia, and Oceania.

Future trials should include larger sample sizes, less-studied variables such as quality of life or readmissions; long-term follow-up; comprehensive explanation of the intervention (frequency, length, and intensity); cost-effectiveness analysis; usability; application of emerging technologies; apps adapted to the age and clinical situation of patients (comorbidity and immobility); and software and hardware improvements such as larger interface fonts or accessible and understandable programs. All these aspects will improve the quality of the trials and help identify the characteristics of the most effective mHealth interventions.

### Conclusions

mHealth technology has a positive effect on patients who have undergone a coronary event in terms of their exercise capacity, performance of physical exercise, adherence to medication, physical and mental quality of life, and hospital readmissions for all causes and cardiovascular causes. More research is required with long-term follow-ups and cost analyses to determine the clinical importance of these findings and to promote their generalization, implementation, and feasibility. A promising future for mHealth technology will be based on the development of apps that are user-friendly and personalized and include motivation and feedback strategies.

## Appendix

Multimedia Appendix 1 Complete search strategy.

Multimedia Appendix 2 Grading of Recommendations, Assessment, Development and Evaluations summary of findings: mobile health versus standard care.

Multimedia Appendix 3 Funnel plots for outcome variables.

Multimedia Appendix 4 Characteristics of included studies.

Multimedia Appendix 5 Forest plots for changes in blood lipids.

Multimedia Appendix 6 Forest plots for changes in systolic blood pressure, diastolic blood pressure, BMI, and waist circumference.

Multimedia Appendix 7 Forest plots for changes in glycated hemoglobin, glucose, heart rate, and smoking cessation.

Multimedia Appendix 8 Forest plots for changes in oxygen consumption peak.

Multimedia Appendix 9 Forest plots for changes in anxiety, depression, and quality of life.

Multimedia Appendix 10 Forest plot for changes in mortality.

Multimedia Appendix 11 Sensitivity analysis.

## Abbreviations

6-MWT: 6-minute walk test
CAD: coronary artery disease
CR: cardiac rehabilitation
CVD: cardiovascular disease
DBP: diastolic blood pressure
HDL: high-density lipoprotein
LDL: low-density lipoprotein
mHealth: mobile health
PRISMA: Preferred Reporting Items for Systematic Reviews and Meta-Analyses
PROSPERO: International Prospective Register of Systematic Reviews
RCT: randomized controlled trial
SBP: systolic blood pressure
