# Supplementary material for: Effectiveness of mHealth Interventions in the Control of Lifestyle and Cardiovascular Risk Factors in Patients After a Coronary Event: Systematic Review and Meta-analysis
Source: JMIR Mhealth Uhealth. 2022 Dec 2;10(12):e39593. doi: 10.2196/39593 (PMC9758644; doi:10.2196/39593)
Supplement: Multimedia Appendix 1 [file mhealth_v10i12e39593_app1.pdf]

**Supplementary table S1.** Complete search strategy	
**#1**	((coronary [Title/Abstract]) OR (infarction [Title/Abstract]) OR ("acute coronary syndrome"[Title/Abstract]) OR (“coronary disease” [Title/Abstract]) OR (cardiac [Title/Abstract]))
**#2**	mhealth [Title/Abstract]) OR (m-health [Title/Abstract])) OR (mobile [Title/Abstract])) OR (smartphone [Title/Abstract])) OR (mobile applications [Title/Abstract]) OR (app [Title/Abstract]))
**#3**	“Randomized controlled trial” [Title/Abstract]) OR (“controlled trial” [Title/Abstract])) OR (“randomized clinical trial” [Title/Abstract])) OR (“randomised controlled trial” [Title/Abstract])) OR (“randomised clinical trial” [Title/Abstract])
	Search (#1 AND #2 AND #3)
