# Supplementary material for: Effectiveness of mHealth Interventions in the Control of Lifestyle and Cardiovascular Risk Factors in Patients After a Coronary Event: Systematic Review and Meta-analysis
Source: JMIR Mhealth Uhealth. 2022 Dec 2;10(12):e39593. doi: 10.2196/39593 (PMC9758644; doi:10.2196/39593)
Supplement: Multimedia Appendix 2 [file mhealth_v10i12e39593_app2.pdf]

Supplementary Table S2. GRADE Summary of Findings: Mhealth vs. Standard care

Outcomes (studies)	Patients (I/C)	Anticipated absolute effects (95% CI)	Relative effect (95%CI)	Quality of evidence	Rated down reasons
Total colesterol (9)	1211 614/597	MD 2.52 lower (8.90 lower to 3.86 higher)		⊕◯◯◯ Very low	a, b, c
LDL colesterol (9)	1207 612/595	MD 3.07 lower (9.50 lower to 3.37 higher)		⊕◯◯◯ Very low	a, b, c
HDL colesterol (7)	943 457/486	MD 0.60 higher (0.33 lower to 1.52 higher)		⊕⊕⊕◯ Moderate	a
Triglycerides (6)	889 439/450	MD 1.65 lower (10.55 lower to 7.25 higher)		⊕⊕⊕◯ Moderate	a, c
Systolic Blood Pressure (13)	2469 1249/1220	MD 0.02 lower (2.39 lower to 2.35 higher)		⊕⊕⊕⊕ High	
Diastolic Blood Pressure (12)	2187 1103/1084	MD 0.62 higher (0.71 lower to 1.95 higher)		⊕⊕◯◯ Low	a, b
Body Mass Index (9)	1986 983/1003	MD 0.02 higher (1.01 lower to 1.05 higher)		⊕⊕⊕⊕ High	
Waist circunference (3)	368 182/186	MD 1.10 lower (4.22 lower to 2.03 higher)		⊕⊕◯◯ Low	a, c
HbA1c (3)	382 189/193	MD 0.16 lower (0.41 lower to 0.10 higher)		⊕◯◯◯ Very low	a, b, c
Glucose (3)	315 145/170	MD 2.44 lower (10.21 lower to 5.34 higher)		⊕⊕◯◯ Low	a, c
Heart rate (3)	494 236/258	MD 2.87 lower (6.33 lower to 0.59 higher)		⊕⊕◯◯ Low	a, c
Smoking cessation (4)	1555 773/782		RD – 0.00 (-0.03 to 0.03)	⊕⊕⊕⊕ High	
6 minutes walk test (4)	1339 669/670	MD 21.64 higher (12.72 higher to 30.55 higher)		⊕⊕⊕◯ Moderate	a, c
Vo2 peak (8)	1512 742/770	MD 0.95 higher (0.40 lower to 2.29 higher)		⊕⊕◯◯ Low	a, b
Physical activity (4)	369 189/180	SMD 0.42 higher (0,04 higher to 0.81 higher)		⊕⊕◯◯ Low	a, b, c
Quality of life. General (9)	1751 867/884	SMD 0.11 higher (0.04 lower to 0.26 higher)		⊕⊕⊕◯ Moderate	a
Quality of life. Physical dimension (5)	620 307/313	SMD 0.26 higher (0.09 higher to 0.44 higher)		⊕⊕⊕⊕ High	
Quality of life. Mental dimension (5)	620 307/313	SMD 0.27 higher (0.06 higher to 0.47 higher)		⊕⊕⊕⊕ High	
Anxiety (4)	612 295/317	SMD 0.08 lower (0.24 lower to 0.07 higher)		⊕⊕◯◯ Low	a, c
Depression (5)	679 328/351	SMD 0.02 lower (0.23 lower to 0.19 higher)		⊕⊕⊕◯ Moderate	a, c
Adherence to medications (3)	507 278/229		RD 0.22 (-0.00 to 0.44)	⊕◯◯◯ Very low	a, b, c
Mortality (3)	2042 1021/1021		RD 0.00 (-0.01 to 0.01)	⊕⊕⊕⊕ High	
Rehospitalization all causes (4)	2081 1041/1040		RD -0.03 (-0.05 to -0.00)	⊕⊕⊕⊕ High	
Rehospitalization cardiovascular causes (6)	1531 769/762		RD -0.04 (-0.07 to -0.00)	⊕⊕⊕◯ Moderate	a

[a] Downgraded because of risks of biases (such as attrition bias, blinding, and other bias).

[b] Downgraded because of inconsistency

[c] Downgraded because of imprecision
