# Supplementary material for: Effectiveness of mHealth Interventions in the Control of Lifestyle and Cardiovascular Risk Factors in Patients After a Coronary Event: Systematic Review and Meta-analysis
Source: JMIR Mhealth Uhealth. 2022 Dec 2;10(12):e39593. doi: 10.2196/39593 (PMC9758644; doi:10.2196/39593)

Supplementary figures S1. Funnel plots for outcome variables.

BMI

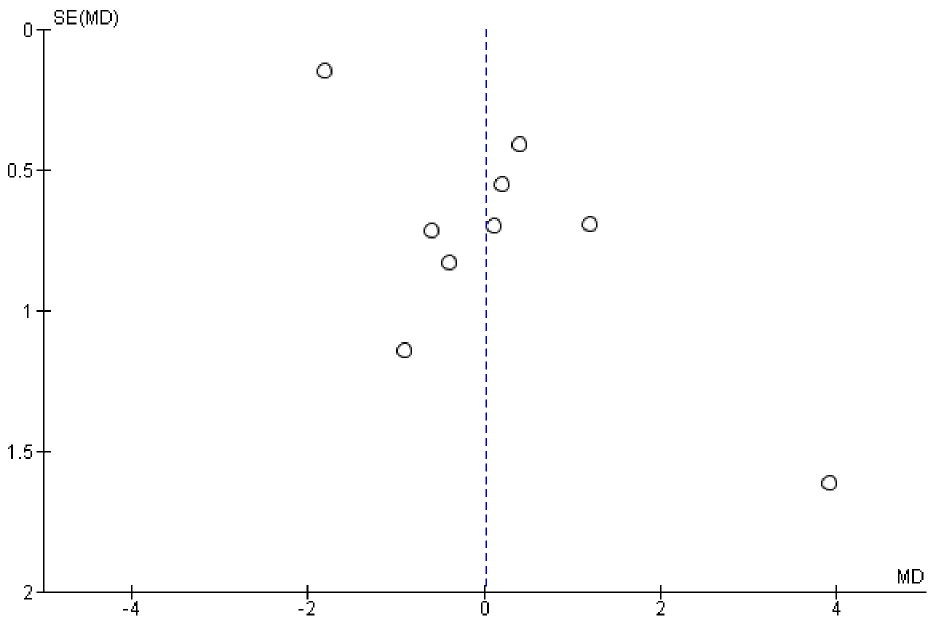

SBP

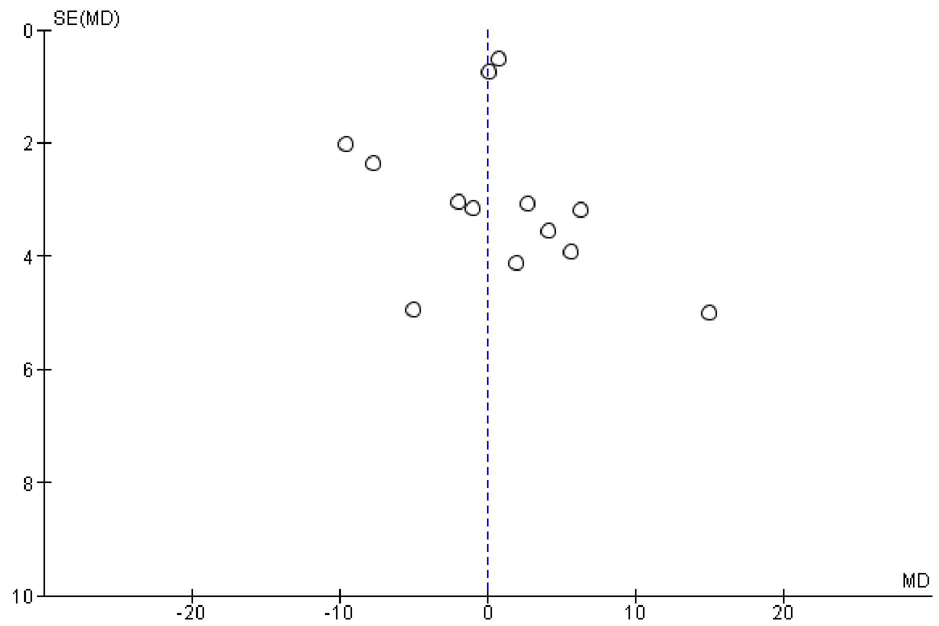

DBP

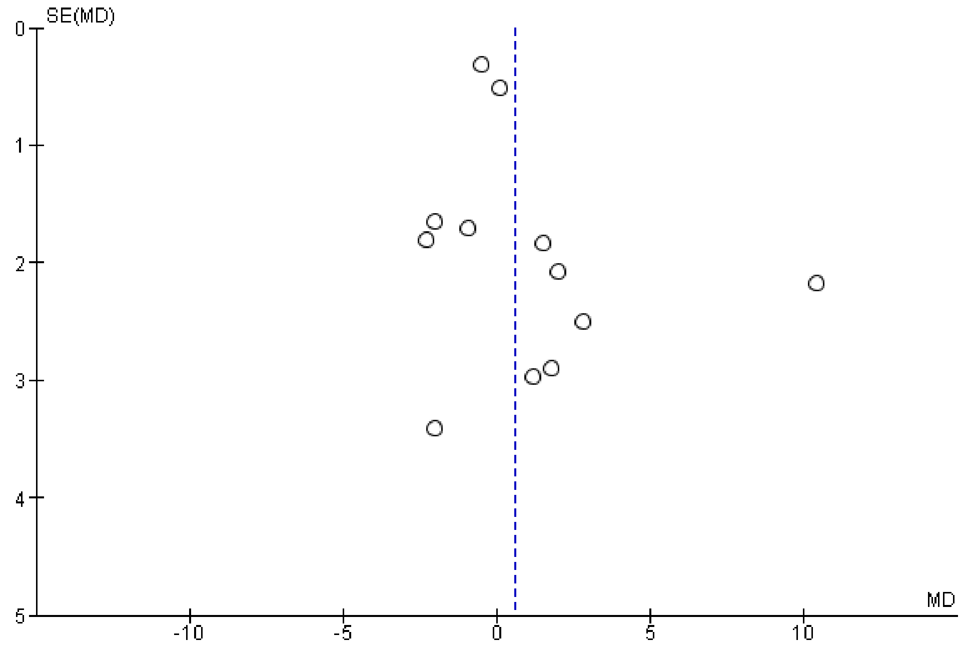

Total cholesterol

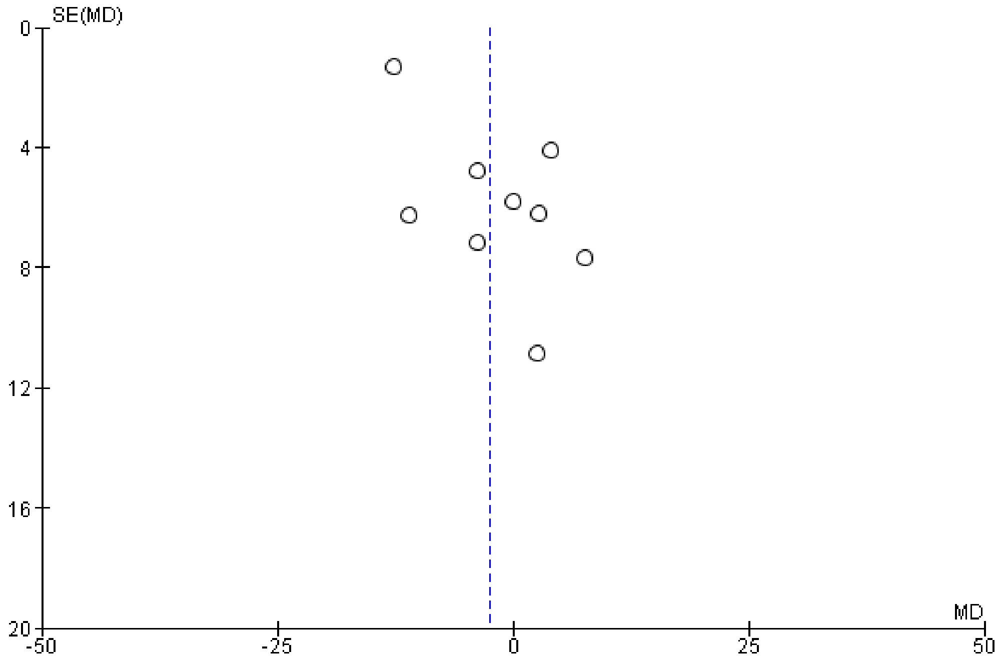

LDL cholesterol

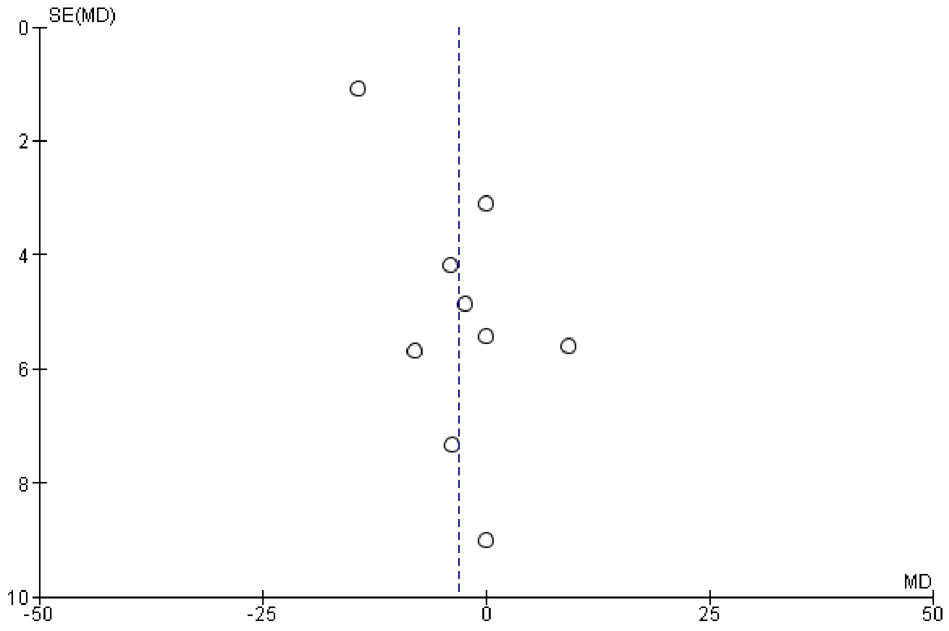

HDL cholesterol

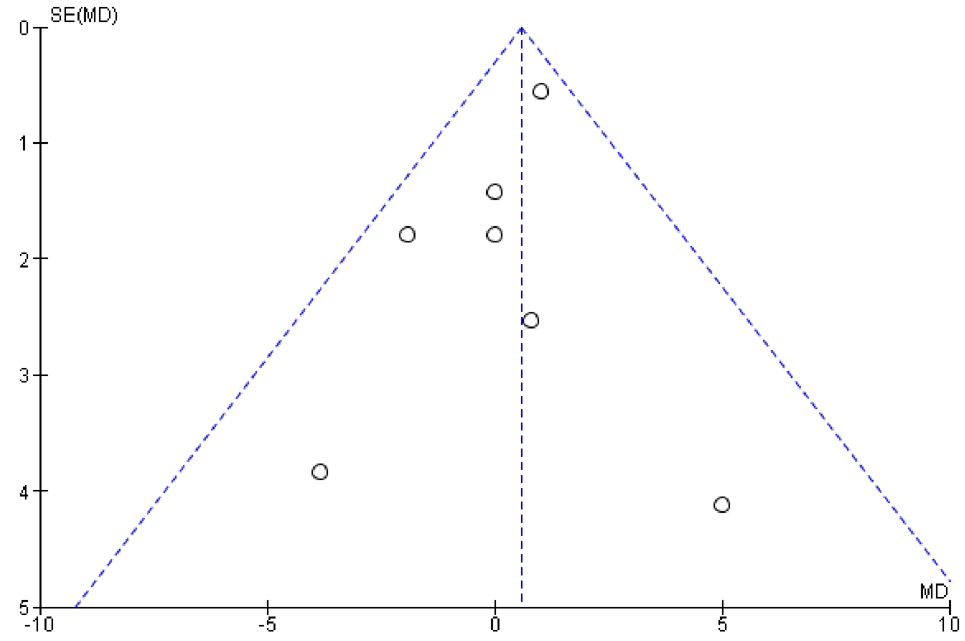

Tryglicerides

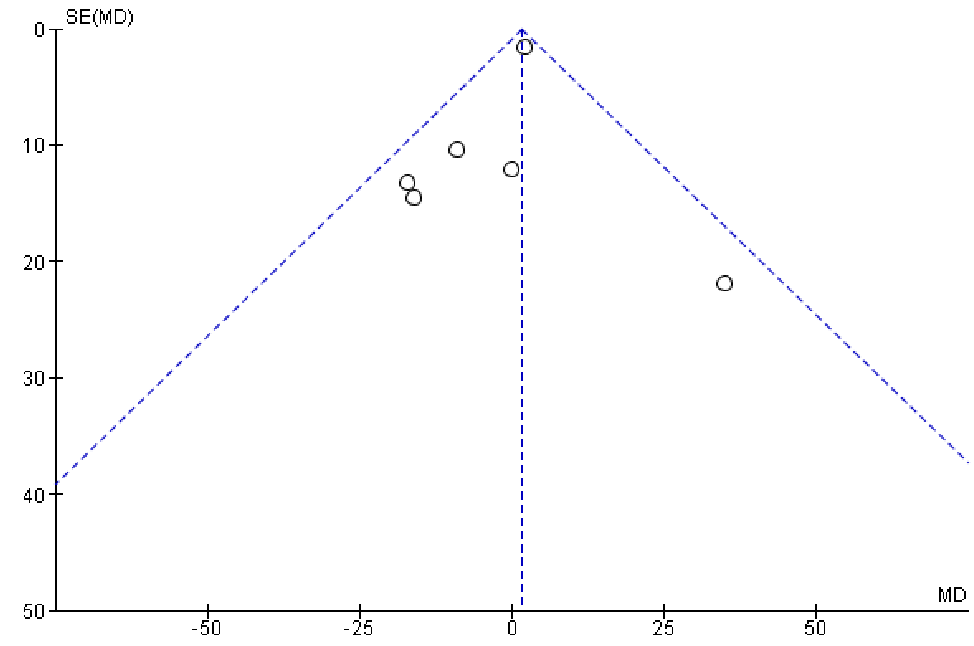

HbA1c

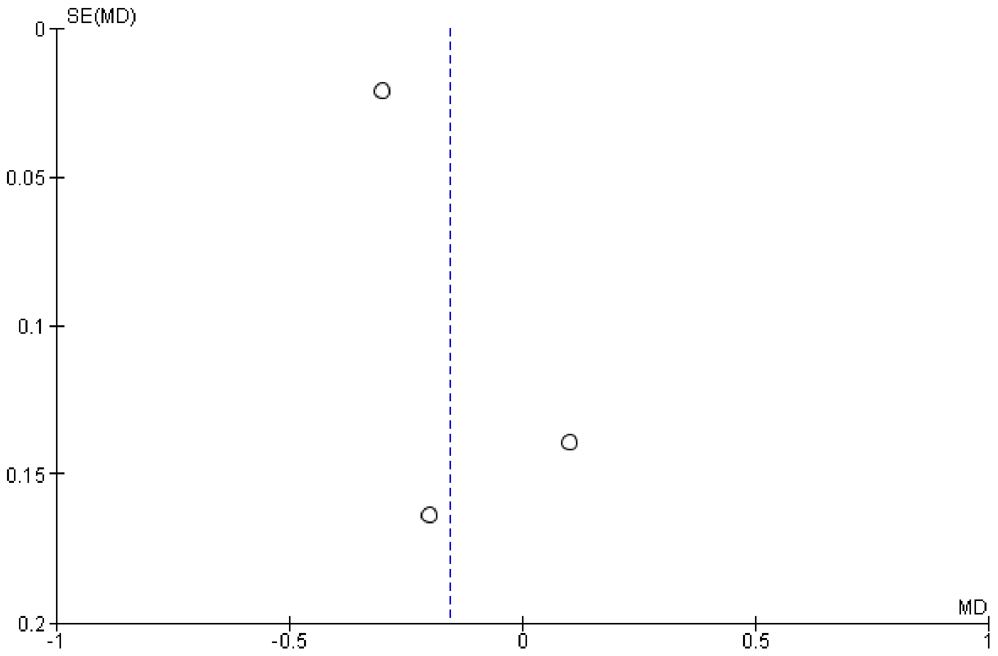

Glucose

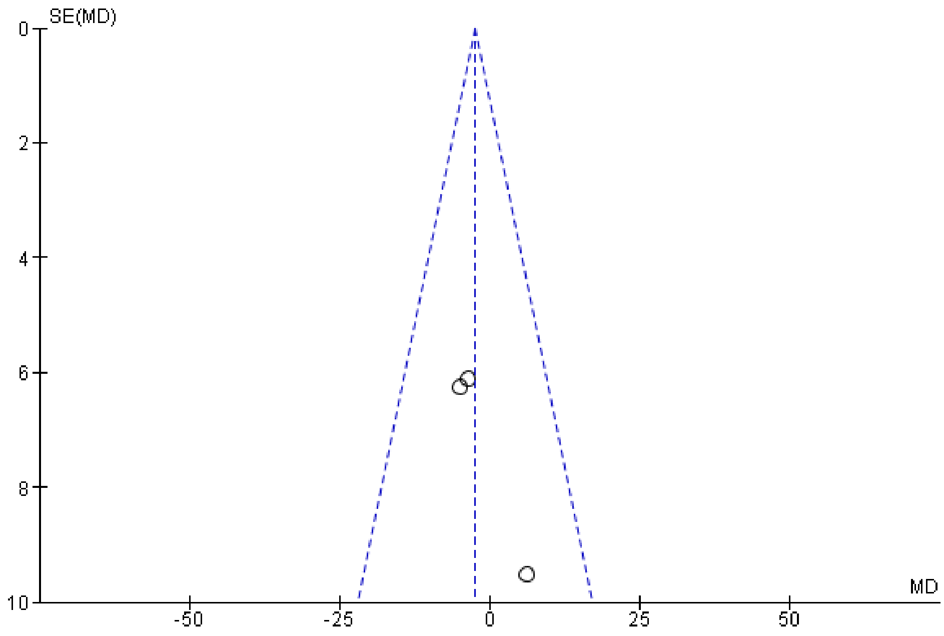

6 minutes walking test

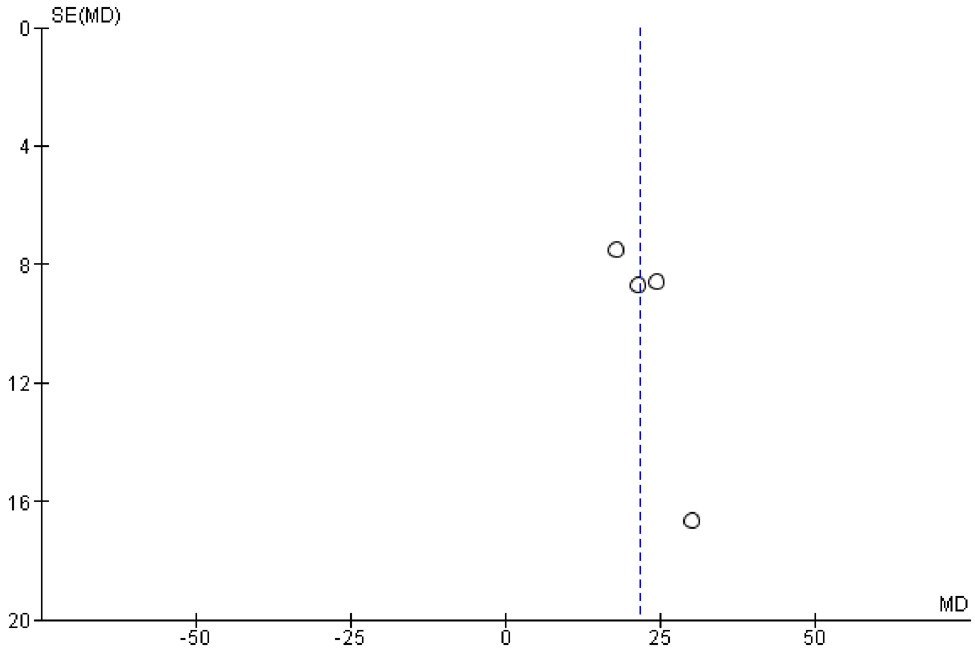

Heart rate

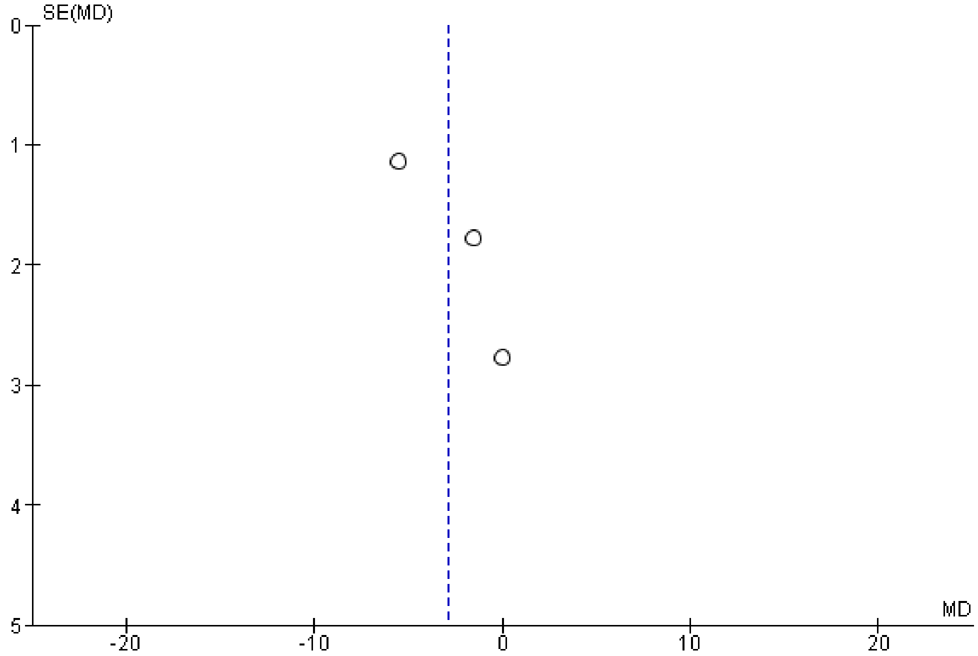

Waist circumference

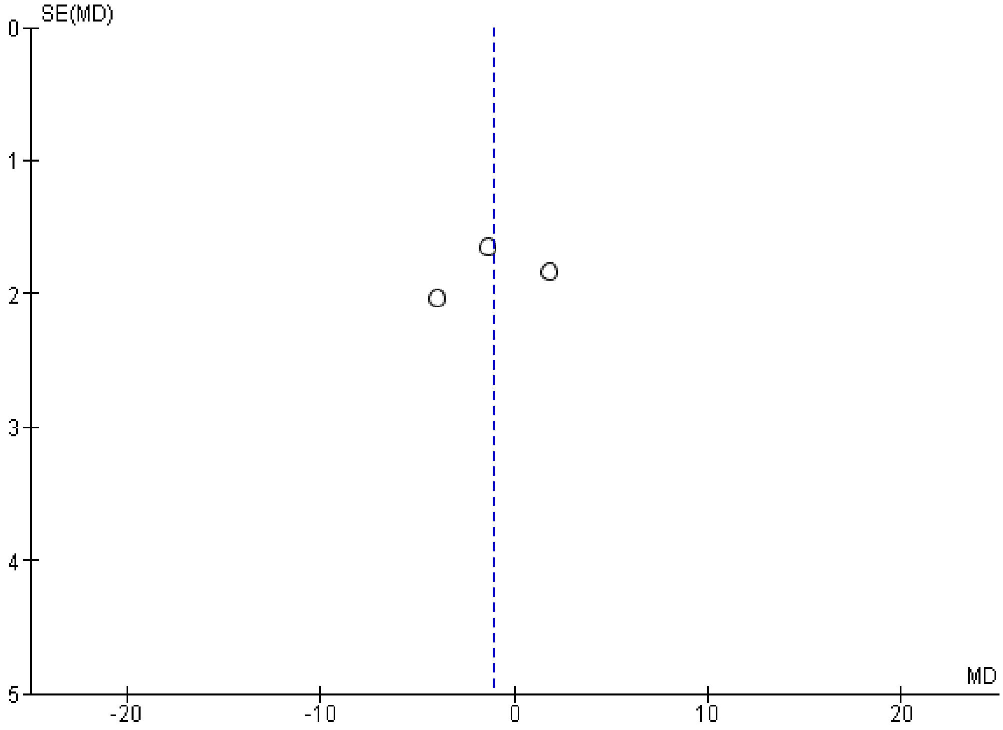

Anxiety

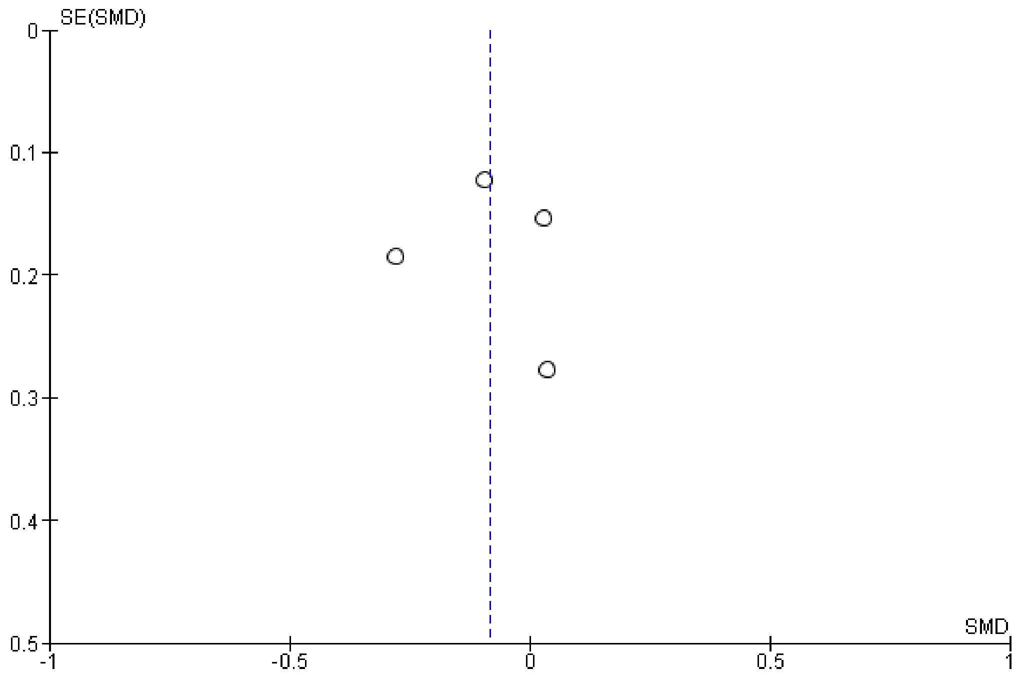

Depression

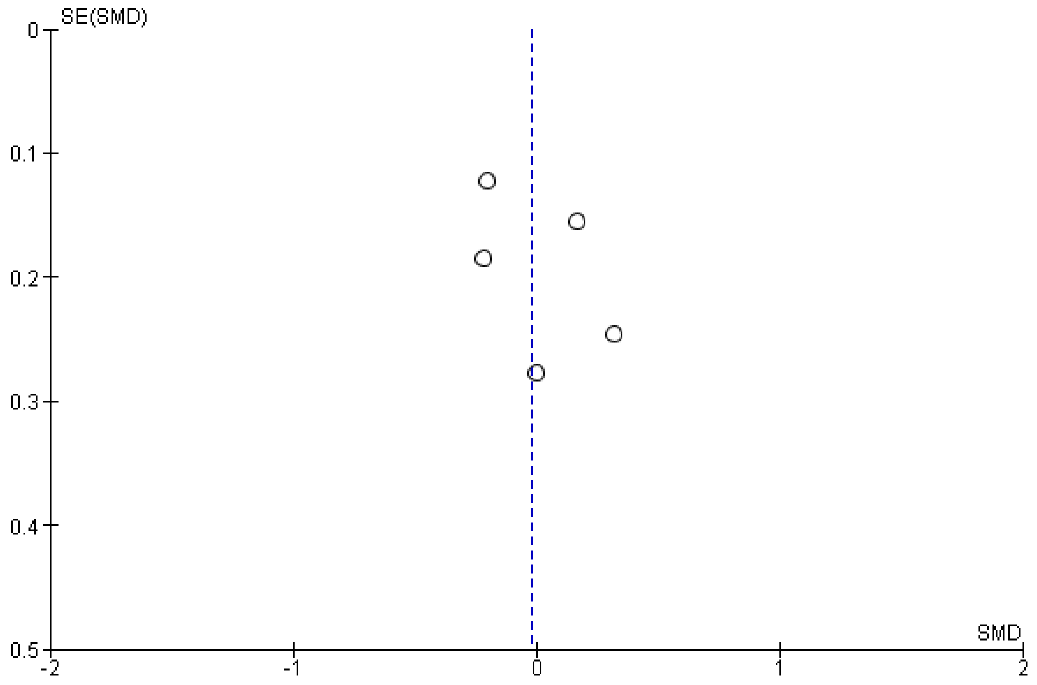

Quality of life. General

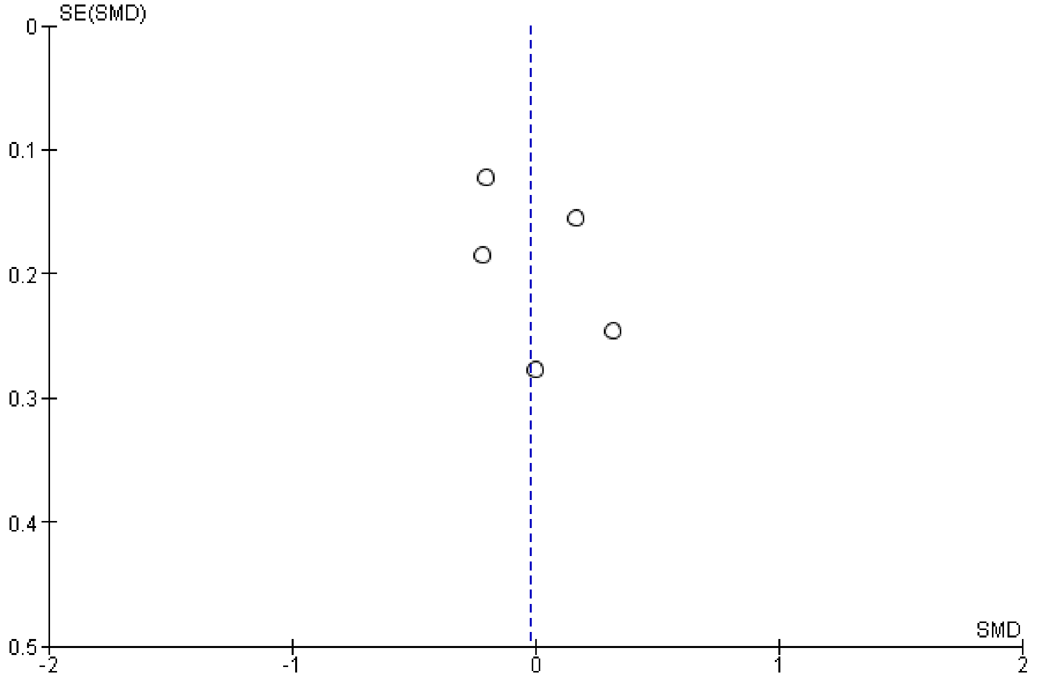

Quality of life. Physical dimensión or physical health

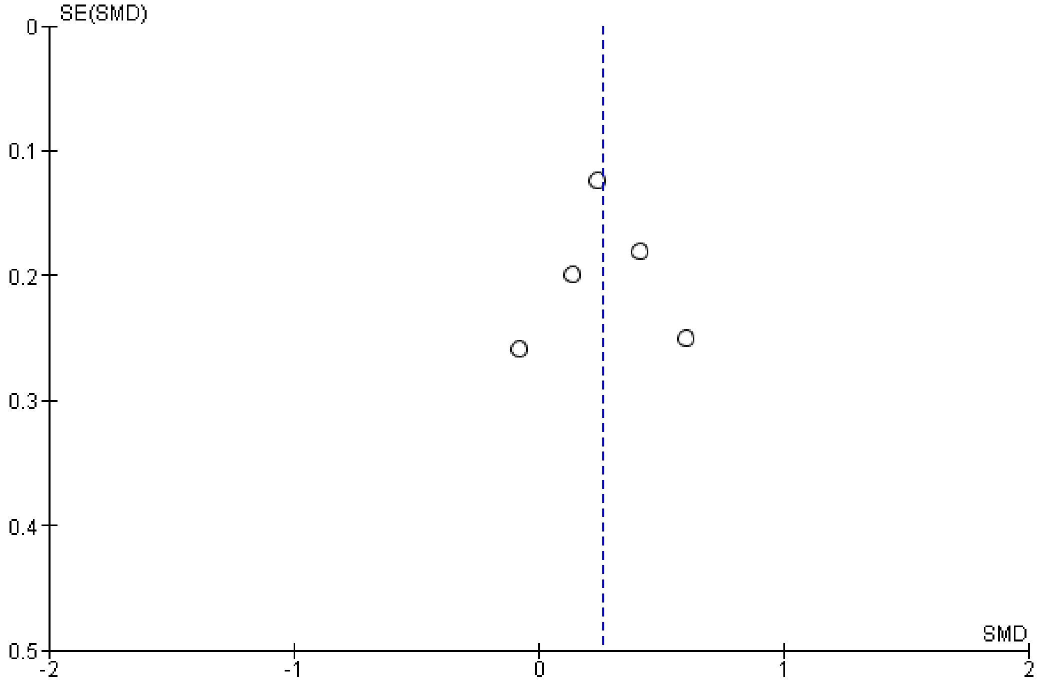

Quality of life. Mental dimension or mental health

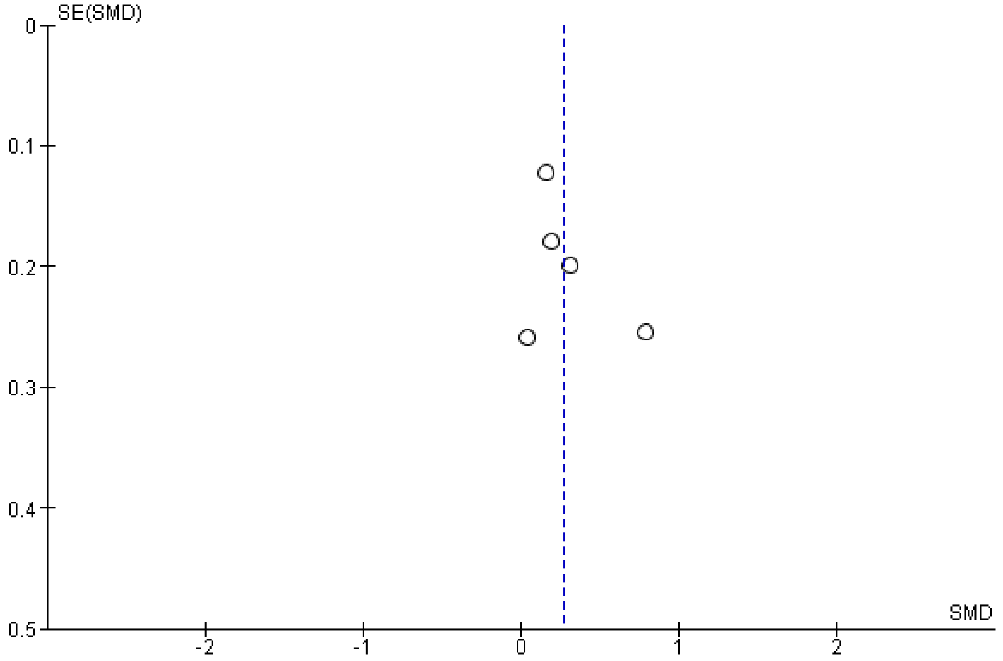

VO2 peak

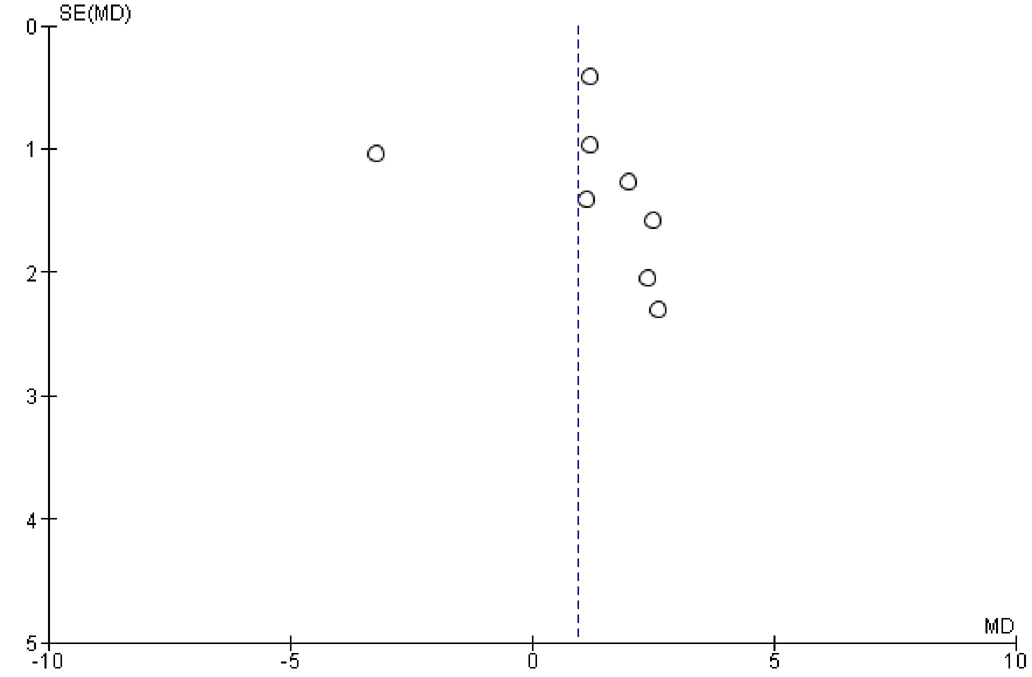

Physical activity

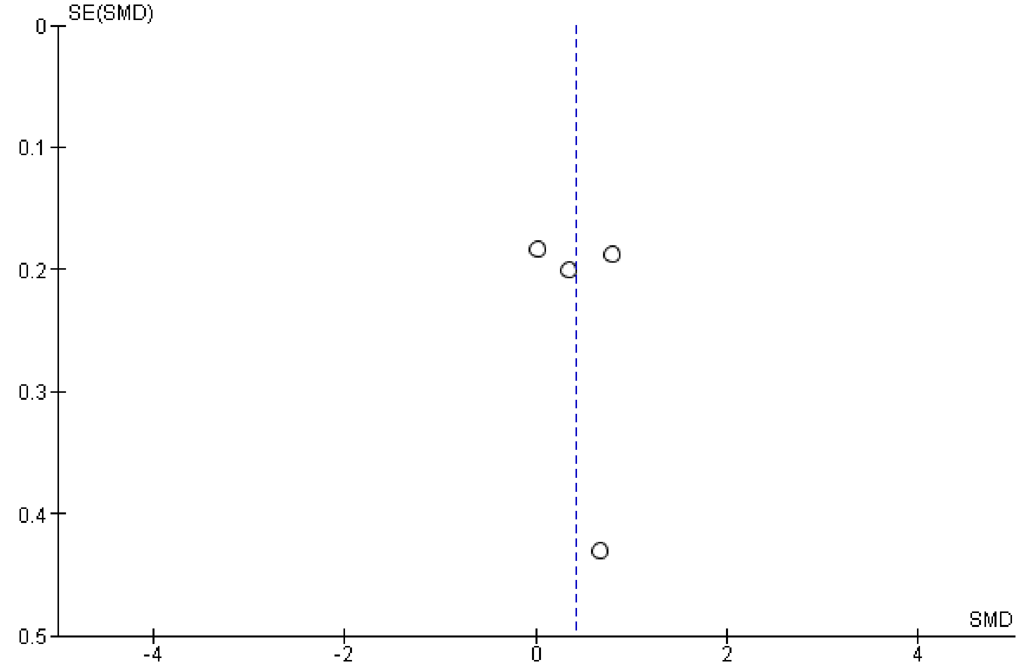

Adherence to medications

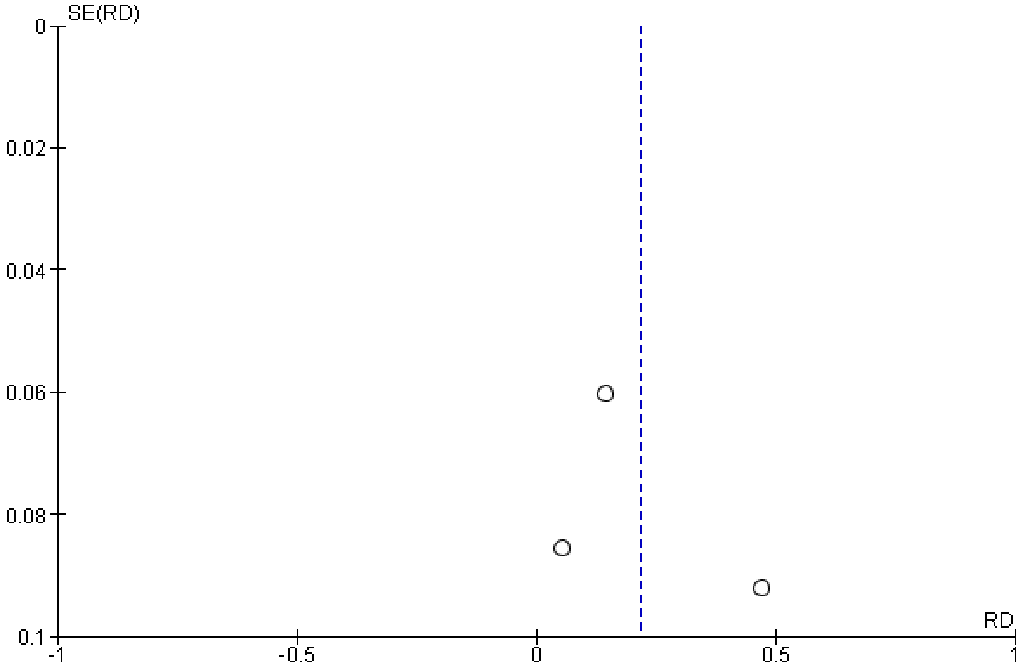

Smoking cessation

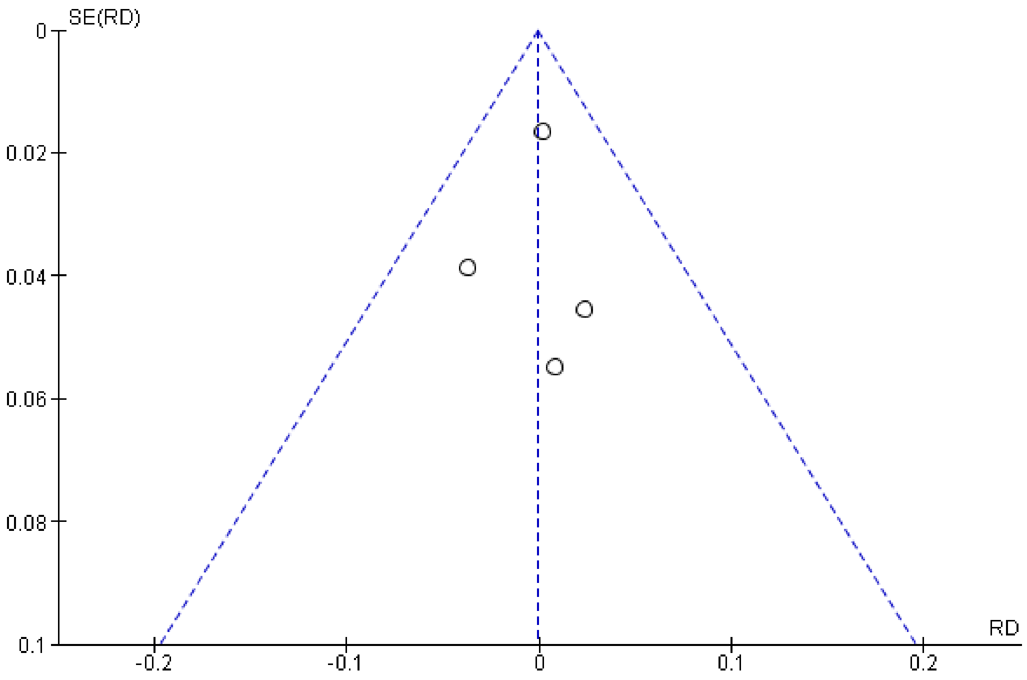

Mortality

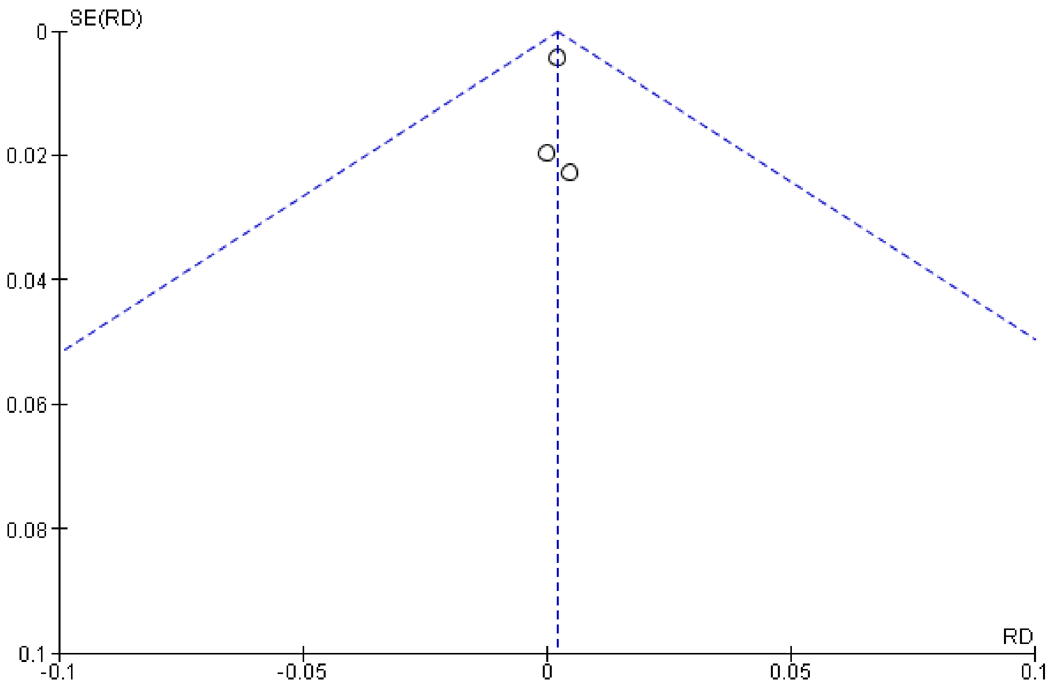

Rehospitalization all causes

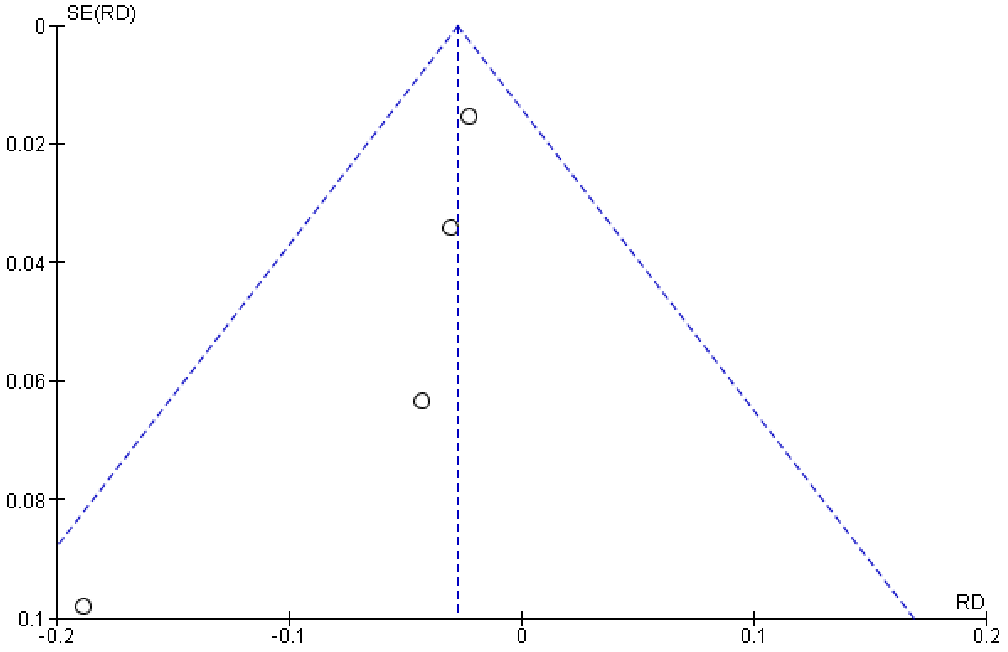

Rehospitalization cardiovascular causes

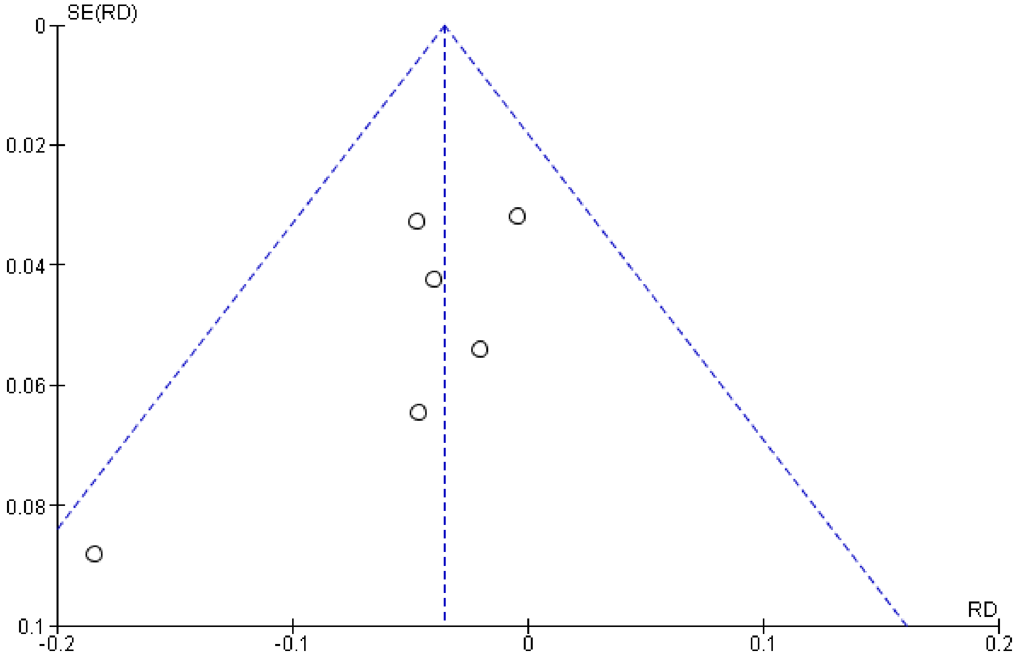

Supplement: Multimedia Appendix 3 [file mhealth_v10i12e39593_app3.pdf]
