# Supplementary material for: Effectiveness of mHealth Interventions in the Control of Lifestyle and Cardiovascular Risk Factors in Patients After a Coronary Event: Systematic Review and Meta-analysis
Source: JMIR Mhealth Uhealth. 2022 Dec 2;10(12):e39593. doi: 10.2196/39593 (PMC9758644; doi:10.2196/39593)
Supplement: Multimedia Appendix 4 [file mhealth_v10i12e39593_app4.pdf]

**Table 1.** Characteristics of included studies.

| Study | Design/ Participants | Inclusion criteria | Intervention and follow-up | Outcomes |
|---|---|---|---|---|
| **Skobel et al. (17)** | - Multicenter<br>**n= 118**<br>Age 58 ± 10<br>**CG n=63**<br>Age 58 (52,67)<br>Lost of follow-up: 21<br>**IG n= 55**<br>Age 60 (50,65)<br>Lost of follow-up: 36 | - CAD (AMI or PCI)<br>- EF ≥ 30%<br>- Willing to exercise | **Intervention:** 6 months<br>**Follow-up:** 6 months<br>**Website:** Smartphone-guided training system with sensor that monitors respiratory rate and electrocardiogram. The medical team provided feedback and adjusted prescriptions.<br>**Professional:** Sport physicians | **Primary**<br>- VO2peak (ml/kg/min)<br>**Secondary**<br>- HDL-c, cLDL-c, total cholesterol<br>- HbA1c, glucose<br>- BMI<br>- Quality of life (EQ-5D).<br>- Anxiety and Depression (HADS-anxiety, HADS-depression) |
| **Choi et al. (18)** | - Unicenter<br>**n= 100**<br>**CG n= 49**<br>Age 56.6± 1.7<br>Lost of follow-up: 5<br>**IG n=51**<br>Age 57.7 ± 1.8<br>Lost of follow-up: 7 | -CVD (atherosclerotic, MI, previous PCI, other)<br>>18 years | **Intervention:** 3 months<br>**Follow-up:** 1, 3 and 6 months<br>**Smartphone application:** Personalised application aimed at reinforcing the Mediterranean diet, with weekly challenges to encourage dietary modification, messaging service.<br>**Professional:** Dietitian and cardiologist | **Primary**<br>- Body weight<br>- Mediterranean diet compliance and satisfaction score (Mediterranean diet score, diet satisfaction score)<br>**Secondary**<br>- SBP, DBP<br>- BMI,<br>- LDL-c, HDL-c, total cholesterol, triglycerides<br>- HbA1c<br>- CRP |
| **Widmer et al. (19)** | - Unicenter<br>**n=80**<br>**CG n=40**<br>Age 63,6±10,9<br>Lost of follow-up: 6 | - ACS (with PCI)<br>> 18 years<br>-Without problem for physical activity | **Intervention:** 3 months<br>**Follow-up:** 3 and 6 months<br>**Smartphone application:** Recording of outcomes in the app for periodic checks, online messaging service. | **Primary**<br>-MACE<br>**Secondary**<br>- Body weight, BMI<br>- SBP, DBP |

| | | | | |
|---|---|---|---|---|
| | **IG n=40**<br>Age 62,5±10,7<br>Lost of follow-up: 3 | | **Professional:** Cardiologist and internist doctor | - Waist circumference<br>- HR<br>- Glucose/HbA1c<br>- LDL-c, HDL-c, total cholesterol, triglycerides<br>- Physical activity (min exe/week)<br>- Diet (food score)<br>- Quality of life(Darmouth QOL)<br>- Stress (stress score)<br>-Acceptability and usability of the app (questionnaire) |
| **Fang et al. (20)** | - Multicenter<br>**n= 80**<br>**CG n=40**<br>Age 61.41 ± 10.169<br>Lost of follow-up: 5<br>**IG n=40**<br>Age 60.24 ± 9.351<br>Lost of follow-up: 6 | - Low-risk patients after PCI<br>-Living with at least one other person | **Intervention:** 1.5 months<br>**Follow-up:** 1.5 months<br>**Smartphone application and website:** Cardiac telerehabilitation at home with real-time monitoring and rapid feedback between the patients and clinicians.<br>**Professional:** Physiotherapist and Doctors | **Primary**<br>-Exercise capacity (6MWT)<br>- SBP, DBP<br>- Anxiety and depression (CDS Score)<br>- Quality of life (SF-36)<br>- Nicotine dependence (FTND score) |
| **Frederix et al. (21)** | - Multicenter<br>**n=140**<br>**CG n=70**<br>Age 61±8<br>Lost of follow-up: 11<br>**IG n=70**<br>Age 61±9<br>Lost of follow-up: 10 | -CAD (with PCI or coronary bypass) / CHF<br>>18 and <80 years | **Intervention:** 6 months<br>**Follow-up:** 12 and 24 months<br>**Website:** Training prescriptions with specific individualised exercises. Recording of the physical activity carried out. Dietary telecoaching with a module for AHT, diabetes mellitus, obesity.<br>**Professional:** Cardiologists | **Primary**<br>- VO2peak (ml/kg/min)<br>**Secondary**<br>- Self-reported physical activity (IPAQ)<br>- Quality of life (HeartQoL),<br>- Cost-effectiveness<br>- HbA1c, glucose<br>- LDL-c, HDL-c, total cholesterol, triglycerides<br>- Cardiovascular readmissions |

| | | | | |
|---|---|---|---|---|
| **Maddison et al. (22)** | - Multicenter<br>**n=171**<br>**CG n=85**<br>Age 59,0±9,5<br>Lost of follow-up: 8<br>**IG n=86**<br>Age 61,4±8,9<br>Lost of follow-up: 10 | - CAD (angina, AMI)<br>-Revascularization, including angioplasty, stent or coronary artery bypass graft within the previous 3–24 months<br>>18 years<br>- Clinically stable | **Intervention:** 6 months<br>**Follow-up:** 6 months<br>**Website + sms:** Regular exercise prescription, behaviour change strategies and technical support with additional information through a website with new weekly content.<br>**Professional:** CR nurses, doctor and physiotherapist | **Primary**<br>- VO2peak (ml/kg/min)<br>**Secondary**<br>-Physical activity (IPAQ-LF).<br>-Self-efficacy (task and barrier scale 0-100)<br>-Quality of life (SF36 v.2 y EQ-5D) |
| **Dorje et al. (23)** | - Multicenter<br>**n=312**<br>**CG n=156**<br>Age 61,9±8,7<br>Lost of follow-up: 25<br>**IG n=156**<br>Age 59,1±9,4<br>Lost of follow-up: 22 | - CAD (with PCI)<br>≥18 years | **Intervention:** 2 months + 4 months<br>**Follow-up:** 2, 6 and 12 months<br>**Website:** Monitoring physical activity with feedback and goal setting; interactive educational modules with avatars; remote blood pressure monitoring and strategies to improve medication adherence.<br>**Professional:** Cardiologist | **Primary**<br>- Exercise capacity (6MWT)<br>**Secondary**<br>- Knowledge of coronary heart disease (knowledge of CHD total score)<br>- BMI<br>- Resting HR<br>- SBP<br>- LDL-c, HDL-c, total cholesterol and triglycerides<br>- Smoking status<br>- Waist circumference<br>- Quality of life (SF-12)<br>- Anxiety (GAD-7 total score)<br>- Depression (PHQ-9 total score)<br>-Cardiac medication adherence (self-reported according to records in the platform)<br>- Utility and acceptability |
| **Hong et al. (24)** | - Multicenter<br>**n= 60** | -CAD (previous MI, coronary artery bypass grafting, PCI or stenosis of | **Intervention:** 3 months<br>**Follow-up:** 3 and 6 months | **Primary**<br>-Self-care and quality of life (PIH scale; WHOQOL- |

| | | | | |
|---|---|---|---|---|
| | 41-60 years (n = 24,40 %)<br>61 -70 years (n = 27, 45 %)<br>**CG n= 30**<br>**IG n= 30** | 50% or more in at least one major epicardial vessel).<br>> 20 years | **Web-based app:** Focused on blood pressure monitoring, self-monitoring behaviour and quality of life. The app has alarm and reminder systems for patients and graphs of their records.<br>**Professional:** Nurses | BREF)<br>- SBP, DBP |
| **Maddison et al. (25)** | - Multicenter<br>**n= 162**<br>**CG n=80**<br>Age 61,5 ± 12,2<br>Lost of follow-up: 11<br>**IG n=82**<br>Age 61,0 ± 13,2<br>Lost of follow-up: 17 | - CAD within the last 6 months (atherosclerosis, angina pectoris, AMI, coronary revascularisation)<br>≥18 year | **Intervention:** 3 months<br>**Follow-up:** 3 and 6 months<br>**Website + smartphone application** of personalised telerehabilitation, with individualised weekly exercise prescription, monitoring and training, behaviour change strategies to promote exercise and regular physical activity.<br>**Professional:** Physiotherapist | **Primary**<br>- VO2peak (ml/kg/min)<br>**Secondary**<br>- HDL-c, LDL-c, total cholesterol and triglycerides<br>- Glucose<br>- Body weight, BMI<br>- Waist/hip circumference<br>- SBP, DBP<br>- Physical activity (min/day)<br>- Motivation to exercise<br>-Quality of life (EQ- 5D index) |
| **Park et al. (26)** | - Multicenter<br>**n= 60**<br>**CG n=28**<br>Age 66.8± 8.7<br>Lost of follow-up: 3<br>**IG n=32**<br>Age 66.7 ± 8.6<br>Lost of follow-up: 6 | - History of CVD: ischaemic heart disease (PCI, bypass, angina and AMI), valvular heart disease, structural heart disease<br>≥ 18 years | **Intervention:** 2 months<br>**Follow-up:** 2 months<br>**Smartphone application** (Movn): To record exercise, weight, BP, HR, medication use. With motivational and educational messages related to CVD management. | **Primary**<br>- Exercise capacity (6MWT)<br>- Steps/day<br>**Secondary**<br>-Depression (PHQ-9) and self-efficacy to maintain exercise (EXSE) |
| **Piotrowicz et al. (27)** | - Multicenter<br>**n= 850** | -HF (ischaemic and non-ischaemic), MI, angioplasty, bypass, valve | **Intervention:** 9 weeks<br>**Follow-up:** 9 weeks, 14 and 26 months | **Primary**<br>- % of days alive and out of the hospital |

| | | | | |
|---|---|---|---|---|
| | **CG n=425**<br>Age 62.2± 10.2<br>Lost of follow-up: 30<br>**IG n=425**<br>Age 62.6 ± 10.8<br>Lost of follow-up: 12 | surgery.<br>- LVEF ≤ 40%<br>- Hospitalised<br>- Clinically stable<br>- No contraindications to undergo CPET | **Website:** Storage of patient data uploaded via mobile phone, monitoring with: a special remote device for supervised exercise training with tele-EKG, a blood pressure device and a body weight scale. Telephone contact for psychological support.<br>**Professional:** Doctors, physiotherapists, nurses and a psychologist. | **Secondary**<br>- MACE<br>- VO2peak (ml/kg/min)<br>- Quality of life (SF-36)<br>- Exercise capacity (6MWT) |
| **Santo et al. (28)** | - Unicenter<br>**n= 163**<br>**CG n= 56**<br>Age 56,8 ±8,64<br>Lost of follow-up: 5<br>**IG n= 107**<br>Age 58,4±9,04<br>Lost of follow-up: 6 | -ACS (MI, unstable or stable angina, PCI or >50% stenosis in at least one major vessel)<br>>18 years | **Intervention:** 3 months<br>**Follow-up:** 3 months<br>**Smartphone application:** Interactive and customisable features, daily medication reminders, which can be postponed, rescheduled and/or marked as a 'taken' or 'missed' dose.<br>**Professional:** Doctors | **Primary**<br>-Adherence to treatment (MMAS-8)<br>**Secondary**<br>-SBP, DBP<br>- LDL-c<br>- Number of missed doses in the last 7 days<br>- Medication knowledge<br>-Acceptability and usefulness of the App (questionnaire) |
| **Snoek et al. (29)** | - Unicenter<br>**n= 122**<br>**CG n=61**<br>Age 59.0 ±10.7<br>Lost of follow-up: 1<br>**IG n=61**<br>Age 60.0 ±8.4<br>Lost of follow-up: 3 | - Have a minimum attendance of 80% in CR and at least one of the following indications for CR: ACS,PCI or coronary artery bypass grafting within three months prior to the start of the CR programme. | **Intervention:** 6 months<br>**Follow-up:** 12 months<br>**Website:** Individualised with physical training records, training history on mobile phone. Telephone contact using motivational interviewing to motivate and stimulate the patient.<br>**Professional:** Nurse | **Primary**<br>- VO2peak (ml/kg/min), FC max<br>**Secondary**<br>- Body weight, BMI<br>- Waist circumference<br>- LDL-c, total cholesterol<br>- Quality of life (Qol, PHQ-9)<br>- Anxiety and depression (HADS)<br>- MACE |

| | | | | |
|---|---|---|---|---|
| **Yu et al. (30)** | - Multicenter<br>**n= 1000**<br>**CG n= 499**<br>Age 57.1± 9.20<br>Lost of follow-up: 10<br>**IG n= 501**<br>Age 57.41 ± 8.99<br>Lost of follow-up: 16 | - Coronary artery bypass<br>>18 years<br>-Have preventive medication prescribed within 2 weeks ofsurgery | **Intervention:** 6 months<br>**Follow-up:** 3 and 6 months<br>**Smartphone application:** With medication records and reminders, in-app messaging service.<br>**Professional:** Nurses | **Primary**<br>- Medication adherence (MMAS-8)<br>**Secondary**<br>- MACE<br>- SBP, DBP<br>- BMI<br>- Smoking |
| **Yudi et al. (31)** | - Multicenter<br>**n= 206**<br>**CG n=103**<br>Age 56.2 ± 10.2<br>Lost of follow-up: 18<br>**IG n= 103**<br>Age 56.8 ± 9.9<br>Lost of follow-up: 17 | -ACS (coronary artery stenosis >50%), or PCI<br>≥18 years | **Intervention:** 2 months<br>**Follow-up:** 2 months<br>**Smartphone application:** With an exercise prescription platform, dynamic CVRF monitoring. Tracking and evaluation of dietary data. Records of prescribed medication within the app. Messaging service with personalised messages. | **Primary**<br>- Exercise capacity (6MWT)<br>**Secondary**<br>- CR adherence and completion<br>- LDL-c, HDL-c, total cholesterol and triglycerides<br>- HbA1c<br>- SBP, DBP<br>- BMI, waist circumference<br>- Smoking<br>-MACE and hospital readmissions (after 2 months)<br>- Depression and anxiety (CDS, HADS)<br>- Quality of life (EQ-5D, SF-36).<br>- Return to work (days) |
| **Lunde et al. (32)** | - Multicenter<br>**n=113**<br>**CG n=56**<br>Age 58,4±8,2<br>Lost of follow-up: 0<br>**IG n=57**<br>Age 59,5±9,1 | - ACS, CAD<br>≥40 years<br>- Completing a CR programme | **Intervention:** 12 months<br>**Follow-up:** 12 months<br>**Smartphone application:** Automatic reminders, task evaluations, weekly goal achievement, short personalised and individualised motivational feedback.<br>**Professional:** Physiotherapist specialising | **Primary**<br>-VO2peak (ml/kg/min)<br>**Secondary**<br>-Exercise performance: Time to exhaustion (sec); Max incline (%) and Max speed (km/h)<br>-Body weight<br>-SBP, DBP |

| | | | | |
|---|---|---|---|---|
| | Lost of follow-up: 2 | | in cardiovascular and pulmonary physiotherapy | - LDL-c, HDL-c, total cholesterol and triglycerides<br>-Exercise habit (exercise sessions (30 min) /week, per year)<br>-Quality of life (HRQL, EQ-5D VAS)<br>-Weekly self-perceived goal achievement (Likert scale 0-100) |
| **Su et al. (33)** | - Unicenter<br>**n= 146**<br>**CG n=73**<br>Age 56.03±7.02<br>Lost of follow-up: 15<br>**IG n=73**<br>Age 55.53 ±7.30<br>Lost of follow-up: 7 | - CHD<br>>18 years<br>-No contraindications for physical activity | **Intervention:** 3 months<br>**Follow-up:** 1.5 and 3 months<br>**Web-based app:** Composed of three platforms: a goal-achievement interface, an experiential learning interface and a health dialogue forum. Participants uploaded goal achievement data on a weekly or daily basis with graphical visualisation and motivational feedback.<br>**Professional:** Nurse | **Primary**<br>- Physical activity (IPAQ, steps/day)<br>- Smoking<br>**Secondary**<br>- Waist circumference, BMI<br>- SBP, DBP<br>- Cardiac self-efficacy (CSES)<br>- Quality of life (MacNew questionnaire)<br>- Anxiety and depression (DASS-21) |
| **Barnason et al. (34)** | - Multicenter<br>**n=50**<br>Age 63 ± 9.3<br>**CG n=25**<br>Lost of follow-up: 4<br>**IG n=25**<br>Lost of follow-up: 3 | - Coronary artery bypass surgery or PCI<br>- Overweight or obese<br>-Participating in a rural CR programme | **Intervention:** 3 months<br>**Follow-up:** 4 and 6 months<br>**Mobile device** (Viterion®): Which included six modules with 36 telehealth sessions addressing calorie goals and dietary portion guidelines + telephone coaching.<br>**Professional:** Nurse | **Primary**<br>- BMI<br>**Secondary**<br>- Physical activity, patient motivation, self-efficacy (CESEI y HHESE scale) |
| **Duscha et al. (35)** | - Multicenter<br>**n = 32**<br>**CG n=9**<br>Age 66,5 ± 7,2 | -CVD (MI with PCI or with coronary bypass / coronary bypass without MI/ PCI without MI/ coronary bypass valve repair/ HF, stable | **Intervention:** 3 months<br>**Follow-up:** 3 months<br>Smartphone application: Using physical activity trackers and health counselling, | **Primary**<br>- VO2peak (ml/kg/min)<br>- Steps/day<br>- Physical activity (min/week) |

| | | | | |
|---|---|---|---|---|
| | Lost of follow-up: 2<br>**IG n=21**<br>Age 59,9±8,1<br>Lost of follow-up: 5 | angina)<br>≥18 year<br>- Completion of 36 CR sessions<br>- Clinical stability<br>- Complete a CPET | with personalised exercise prescription.<br>**Professional:** Nutritionists, exercise physiologists, nurses and health educators. | |
| **Johnston et al. (36)** | - Multicenter<br>**n= 174**<br>**CG n= 83**<br>Age 58,4<br>Lost of follow-up: 6<br>**IG n=91**<br>Age 56,8<br>Lost of follow-up: 6 | - MI<br>>18 years<br>-With treatment initiated in-hospital with ticagrelor 90 mg 2/day one year | **Intervention:** 1 month<br>**Follow-up:** 6 months<br>**Web-based app:** Installed on the smartphone with an electronic diary focusing on medication adherence and secondary prevention education modules. Educational messages according to reported records.<br>**Professional:** Doctor and Nurse | **Primary**<br>Adherence to treatment (electronic diary) (MARS-5)<br>**Secondary**<br>- BMI<br>- SBP<br>- LDL-c<br>- Physical activity level (min/week)<br>- App Usability (SUS)<br>- Smoking cessation<br>-Quality of life (EQ- 5D VAS) |
| **Song et al. (37)** | - Multicenter<br>**n= 106**<br>**CG n=53**<br>Age 54.83 ± 9.13<br>Lost of follow-up: 5<br>**IG n=53**<br>Age 54.17 ± 8.76<br>Lost of follow-up: 5 | - Stable CHD<br>≤ 75 years<br>-Without physical or mental disorders affecting exercise | **Intervention:** 6 months<br>**Follow-up:** 6 months<br>Remote **monitoring software** installed on **smartphones**: Rehabilitation with tele-monitored exercises. Weekly communication through text messaging and phone calls.<br>**Professional:** Doctors | **Primary**<br>- VO2peak (ml/kg/min)<br>**Secondary**<br>- Improved exercise tolerance (CPET)<br>- HDL-c, LDL-c, triglycerides<br>- Glucose<br>- MACE |
| **Treskes et al. (38)** | - Unicenter<br>**n=200**<br>**CG n=100** | - MI<br>> 18 years<br>- BMI <35 kg/m2 | **Intervention:** 12 months<br>**Follow-up:** 1, 6 and 12 months<br>**Smartphone application:** Daily recording | **Primary**<br>- SBP and DBP control<br>**Secondary** |

| | | | | |
|---|---|---|---|---|
| | Age 59,1 ± 30,3<br>Lost of follow-up: 8<br>**IG n=100**<br>Age 60,1 ± 34,6<br>Lost of follow-up: 12 | | and review of variables, contact if BP is higher than recommended or arrhythmias. Management of 4 devices + Smartphone (BP monitor, pedometer, weighing scale and ECG device).<br>**Professional:** Nurse specialised and cardiologist | -Patient's Satisfaction (Patient's Satisfaction Questionnaire)<br>- MACE (one year after MI) |
| **Garmendia et al. (39)** | - Unicenter<br>**n=90**<br>**CG n=44**<br>Age 62±10<br>Lost of follow-up: 0<br>**IG n=46**<br>Age 64±10<br>Lost of follow-up: 0 | -ACS hospitalised (with and without ST elevation)<br>> 18 years | **Intervention:** 3 months<br>**Follow-up:** 3 months<br>**Smartphone application** (MyTherapy®): Where the medical team installs a list of prescribed medication with alarms as a reminder.<br>**Professional:** Doctor | **Primary**<br>- Adherence to treatment (MMAS-8) |

**ACS:** acute coronary syndrome; **AMI:** acute myocardial infarction; **BMI:** body mass index; **CAD:** coronary artery disease; **CDS:** Calgary Depression Scale; **CESEI:** Cardiac Exercise Self-Efficacy Instrument; **CG:** Control Group; **CHD:** coronary heart disease; **CHF:** chronic heart failure; **CSES:** Cardiac Self-efficacy Scale; **CR:** cardiac rehabilitation; **C-RP:** c-reactive protein; **CPET:** cardiopulmonary exercise test; **CVD:** cardiovascular disease; **CVRF:** cardiovascular risk factors; **DASS-21:** Depression Anxiety Stress Scale 21; **DBP:** diastolic blood pressure; **EKG:** electrocardiogram; **EQ-5D VAS:** European Quality of Life-5 Dimensions, visual analog scale; **EXSE:** Exercise Self-Efficacy Scale; **FTND:** Fagerström Test for Nicotine Dependence; **GAD-7:** Generalized Anxiety Disorder; **HADS:** Hospital Anxiety and Depression Scale; **HbA1c:** glycosylated hemoglobin; **HBP:** high blood pressure; **HDL-c:** high-density lipoprotein cholesterol; **HF:** heart failure; **HHESE:** Heart Healthy Eating Self-Efficacy; **HR:** heart rate; **HRQL:** Health-Related Quality of Life; **IG:** Intervention Group; **IPAQ:** International Physical Activity Questionnaire; **LDL-c:** low- density lipoprotein cholesterol; **LVEF:** left ventricular ejection fraction; **MACE:** major adverse cardiac events; **MacNew:** MacNew Heart Disease Health-related Quality of Life; **MARS-5:** Medication Adherence Report Scale; **MI:** myocardial infarction; **MMAS-8:** Morisky Medication Adherence Scale-8; **PCI:** percutaneous coronary intervention; **PHQ-9:** Patient Health Questionnaire; **PIH:** Partners in Health; **QoL;** Quality of Life; **SBP:** systolic blood pressure; **SF-12:** 12-item short form health survey; **SF-36:** 36-item health survey; **SUS:** system usability scale; **Vo2 peak**: Peak oxygen uptake; **WHOQoL-BREF:** World Health Organization Quality of Life; **6MWT:** six minutes walk test.
