# Supplementary material for: Effectiveness of mHealth Interventions in the Control of Lifestyle and Cardiovascular Risk Factors in Patients After a Coronary Event: Systematic Review and Meta-analysis
Source: JMIR Mhealth Uhealth. 2022 Dec 2;10(12):e39593. doi: 10.2196/39593 (PMC9758644; doi:10.2196/39593)

Supplementary figures S2. Forest plots for changes in blood lipids.

Total cholesterol

	mHealth			Control				Mean Difference
Study or Subgroup	Mean	SD	Total	Mean	SD	Total	Weight	IV, Random, 95% CI
Choi BG et al., 2019	159.3	6.1	44	172	6.4	44	17.0%	-12.70 [-15.31, -10.09]
Dorje T et al, 2019	139.2	38.7	134	135.3	27.1	131	13.7%	3.90 [-4.13, 11.93]
Frederix I et al, 2017	143	28	62	154	41	64	10.6%	-11.00 [-23.23, 1.23]
Lunde P et al., 2020	158.4	38.7	48	150.8	38.7	54	8.9%	7.60 [-7.45, 22.65]
Maddison R et al., 2019	140	38	68	137.3	35.6	72	10.6%	2.70 [-9.51, 14.91]
Santo K et al., 2019	139.2	34.8	98	139.2	32.5	49	11.2%	0.00 [-11.41, 11.41]
Skobel E et al., 2017	151.8	40	18	149.2	33.7	39	5.9%	2.60 [-18.69, 23.89]
Snoek et al., 2021	162.16	34.75	59	166.02	42.47	59	9.5%	-3.86 [-17.86, 10.14]
Yudi MB et al., 2020	131.5	27.1	83	135.3	34.8	85	12.6%	-3.80 [-13.22, 5.62]
**Total (95% CI)**			**614**			**597**	**100.0%**	**-2.52 [-8.90, 3.86]**

Heterogeneity: Tau² = 60.71; Chi² = 31.13, df = 8 (P = 0.0001); I² = 74%
Test for overall effect: Z = 0.77 (P = 0.44)

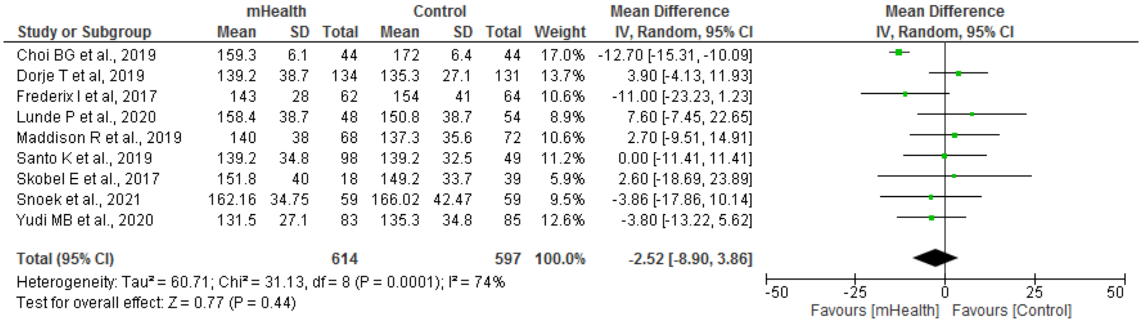

LDL cholesterol

	mHealth			Control				Mean Difference
Study or Subgroup	Mean	SD	Total	Mean	SD	Total	Weight	IV, Random, 95% CI
Choi BG et al., 2019	84.1	4.9	44	98.3	5.4	44	15.1%	-14.20 [-16.35, -12.05]
Dorje T et al, 2019	69.6	27.1	134	69.6	23.2	131	13.5%	0.00 [-6.07, 6.07]
Frederix I et al, 2017	74	26	62	82	37	64	10.5%	-8.00 [-19.14, 3.14]
Lunde P et al., 2020	85.1	38.7	48	88.9	34.8	54	8.7%	-3.80 [-18.15, 10.55]
Maddison R et al., 2019	75.4	37.5	68	66.1	27.8	72	10.6%	9.30 [-1.69, 20.29]
Santo K et al., 2019	62.6	29	98	64.9	27.1	49	11.5%	-2.30 [-11.82, 7.22]
Skobel E et al., 2017	77.5	31.7	16	77.5	25.8	37	7.1%	0.00 [-17.62, 17.62]
Snoek et al., 2021	88.8	23.03	59	88.8	34.75	59	10.8%	0.00 [-10.64, 10.64]
Yudi MB et al., 2020	65.7	27.1	83	69.6	27.1	85	12.3%	-3.90 [-12.10, 4.30]
**Total (95% CI)**			**612**			**595**	**100.0%**	**-3.07 [-9.50, 3.37]**

Heterogeneity: Tau² = 70.32; Chi² = 46.78, df = 8 (P < 0.00001); I² = 83%
Test for overall effect: Z = 0.93 (P = 0.35)

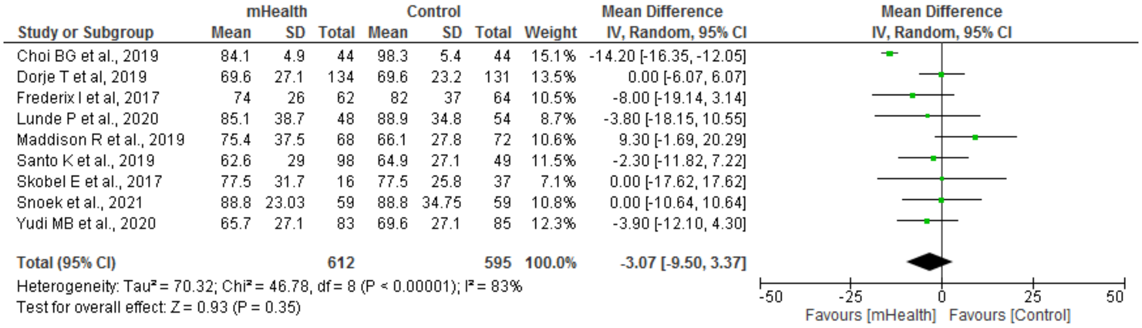

HDL cholesterol

	mHealth			Control				Mean Difference
Study or Subgroup	Mean	SD	Total	Mean	SD	Total	Weight	IV, Random, 95% CI
Choi BG et al., 2019	55.3	2.8	44	54.3	2.5	44	69.1%	1.00 [-0.11, 2.11]
Dorje T et al, 2019	46.4	11.6	134	46.4	11.6	131	10.9%	0.00 [-2.79, 2.79]
Frederix I et al, 2017	47	11	62	48.9	9	64	6.9%	-1.90 [-5.42, 1.62]
Lunde P et al., 2020	50.27	19.3	48	54.1	19.3	54	1.5%	-3.83 [-11.33, 3.67]
Maddison R et al., 2019	44.5	15.5	68	43.7	14.3	72	3.5%	0.80 [-4.15, 5.75]
Skobel E et al., 2017	47.9	15.9	18	42.9	10.2	36	1.3%	5.00 [-3.07, 13.07]
Yudi MB et al., 2020	42.5	11.6	83	42.5	11.6	85	6.9%	0.00 [-3.51, 3.51]
**Total (95% CI)**			**457**			**486**	**100.0%**	**0.60 [-0.33, 1.52]**

Heterogeneity: Tau² = 0.00; Chi² = 5.22, df = 6 (P = 0.52); I² = 0%
Test for overall effect: Z = 1.27 (P = 0.21)

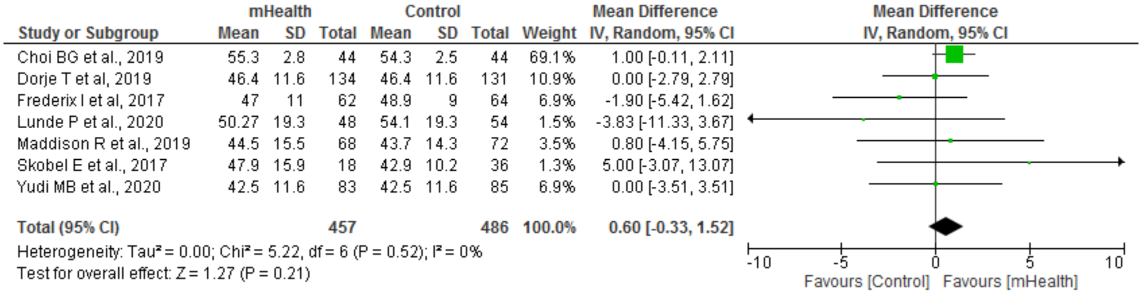

Triglycerides

	mHealth			Control				Mean Difference
Study or Subgroup	Mean	SD	Total	Mean	SD	Total	Weight	IV, Random, 95% CI
Choi BG et al., 2019	99.8	8.5	44	97.5	7.3	44	52.4%	2.30 [-1.01, 5.61]
Dorje T et al, 2019	132.7	115	134	132.7	79.6	131	11.2%	0.00 [-23.77, 23.77]
Frederix I et al, 2017	118	65	62	135	83	64	9.7%	-17.00 [-42.99, 8.99]
Lunde P et al., 2020	140	140	48	105	61.2	54	4.0%	35.00 [-7.84, 77.84]
Maddison R et al., 2019	130.9	71.7	68	146.9	98.2	72	8.4%	-16.00 [-44.37, 12.37]
Yudi MB et al., 2020	115	53	83	123.9	79.6	85	14.2%	-8.90 [-29.30, 11.50]
**Total (95% CI)**			**439**			**450**	**100.0%**	**-1.65 [-10.55, 7.25]**

Heterogeneity: Tau² = 36.46; Chi² = 6.99, df = 5 (P = 0.22); I² = 28%
Test for overall effect: Z = 0.36 (P = 0.72)

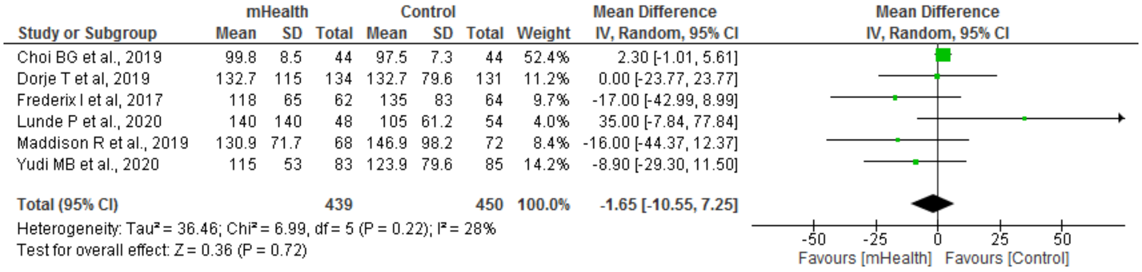

Supplement: Multimedia Appendix 5 [file mhealth_v10i12e39593_app5.pdf]
