# Supplementary material for: Effectiveness of mHealth Interventions in the Control of Lifestyle and Cardiovascular Risk Factors in Patients After a Coronary Event: Systematic Review and Meta-analysis
Source: JMIR Mhealth Uhealth. 2022 Dec 2;10(12):e39593. doi: 10.2196/39593 (PMC9758644; doi:10.2196/39593)

Supplementary figures S2. Forest plots for changes in systolic blood pressure, diastolic blood pressure, body mass index and waist circumference.

Systolic blood pressure

Study or Subgroup	mHealth Mean	mHealth SD	mHealth Total	Control Mean	Control SD	Control Total	Weight	Mean Difference IV, Random, 95% CI
Choi BG et al., 2019	129.5	2.5	44	128.7	2.5	44	13.5%	0.80 [-0.24, 1.84]
Dorje T et al, 2019	122.5	13.2	134	132	19	131	10.0%	-9.50 [-13.45, -5.55]
Fang J et al., 2018	135.2	16.1	33	129.6	16.1	34	5.6%	5.60 [-2.11, 13.31]
Frederix I et al, 2017	131	25	62	129	21	64	5.3%	2.00 [-6.07, 10.07]
Hong et al., 2021	129.17	13.85	30	122.87	10.56	30	7.1%	6.30 [0.07, 12.53]
Lunde P et al., 2020	140	32	60	145	21	59	4.2%	-5.00 [-14.71, 4.71]
Maddison R et al., 2019	135.4	18.2	65	132.7	17.4	69	7.3%	2.70 [-3.34, 8.74]
Santo K et al., 2019	125	17	101	126	19.1	51	7.1%	-1.00 [-7.20, 5.20]
Skobel E et al., 2017	139	19	19	124	16	42	4.1%	15.00 [5.18, 24.82]
Snoek et al., 2021	127	16	59	129	17	59	7.4%	-2.00 [-7.96, 3.96]
Su et al., 2021	118.47	11.24	66	126.2	14.59	58	9.1%	-7.73 [-12.36, -3.10]
Yu C et al., 2020	124.7	11.3	493	124.6	12.2	494	13.2%	0.10 [-1.37, 1.57]
Yudi MB et al., 2020	119.3	22.4	83	115.2	23.7	85	6.3%	4.10 [-2.87, 11.07]
**Total (95% CI)**			**1249**			**1220**	**100.0%**	**-0.02 [-2.39, 2.35]**

Heterogeneity: Tau² = 10.52; Chi² = 53.85, df = 12 (P < 0.00001); I² = 78%
Test for overall effect: Z = 0.02 (P = 0.99)

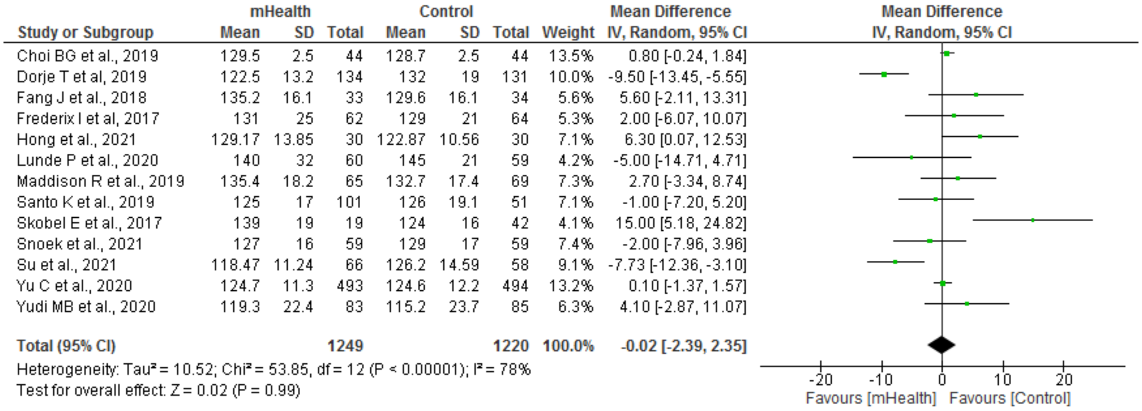

Diastolic blood pressure

Study or Subgroup	mHealth Mean	mHealth SD	mHealth Total	Control Mean	Control SD	Control Total	Weight	Mean Difference IV, Random, 95% CI
Choi BG et al., 2019	78.7	1.5	44	79.2	1.5	44	18.5%	-0.50 [-1.13, 0.13]
Fang J et al., 2018	83.2	12.7	33	81.4	10.9	34	4.3%	1.80 [-3.87, 7.47]
Frederix I et al, 2017	77	21	62	79	17	64	3.3%	-2.00 [-8.68, 4.68]
Hong et al., 2021	71.63	10.81	30	68.83	8.35	30	5.3%	2.80 [-2.09, 7.69]
Lunde P et al., 2020	86	10	48	84	11	54	6.9%	2.00 [-2.08, 6.08]
Maddison R et al., 2019	79.2	10.5	65	77.7	10.8	69	8.0%	1.50 [-2.11, 5.11]
Santo K et al., 2019	80.2	8.7	101	81.1	10.5	51	8.7%	-0.90 [-4.24, 2.44]
Skobel E et al., 2017	83	7	19	72.6	9.5	42	6.5%	10.40 [6.14, 14.66]
Snoek et al., 2021	79	9	59	81	9	59	9.0%	-2.00 [-5.25, 1.25]
Su et al., 2021	73.63	9.23	66	75.9	10.71	58	8.1%	-2.27 [-5.81, 1.27]
Yu C et al., 2020	79.8	7.9	493	79.7	8.2	494	17.4%	0.10 [-0.90, 1.10]
Yudi MB et al., 2020	83.3	18.2	83	82.1	20.3	85	4.1%	1.20 [-4.63, 7.03]
**Total (95% CI)**			**1103**			**1084**	**100.0%**	**0.62 [-0.71, 1.95]**

Heterogeneity: Tau² = 2.38; Chi² = 32.02, df = 11 (P = 0.0008); I² = 66%
Test for overall effect: Z = 0.92 (P = 0.36)

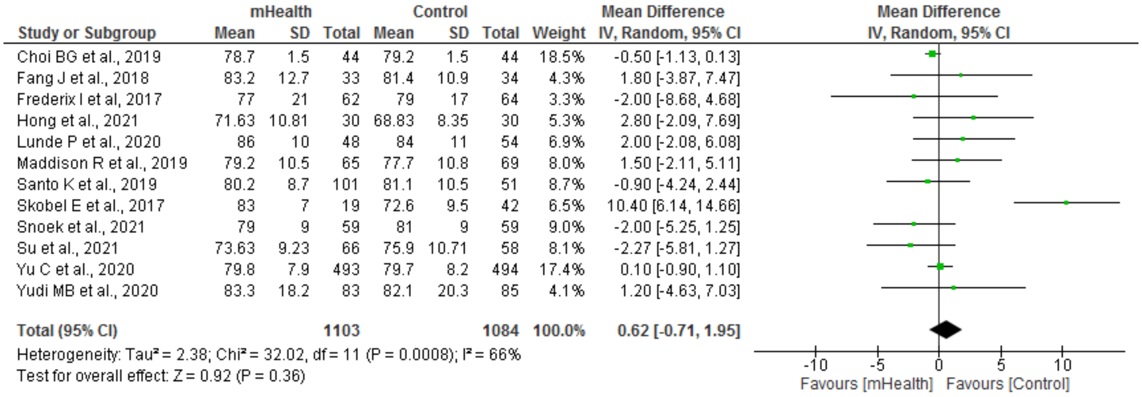

Body mass index

Study or Subgroup	mHealth Mean	mHealth SD	mHealth Total	Control Mean	Control SD	Control Total	Weight	Mean Difference IV, Random, 95% CI
Barnason S et al., 2019	35.2	6	20	31.3	4.1	21	6.1%	3.90 [0.74, 7.06]
Choi BG et al., 2019	28.7	0.7	44	30.5	0.7	44	14.3%	-1.80 [-2.09, -1.51]
Dorje T et al, 2019	24.9	3.5	134	24.5	3.2	131	13.3%	0.40 [-0.41, 1.21]
Maddison R et al., 2019	29.1	4.5	65	27.9	3.4	69	11.6%	1.20 [-0.16, 2.56]
Skobel E et al., 2017	27.3	4.5	19	28.2	3.2	42	8.6%	-0.90 [-3.14, 1.34]
Snoek et al., 2021	27.9	3.8	59	28.5	4	59	11.4%	-0.60 [-2.01, 0.81]
Su et al., 2021	24.7	3.6	66	24.5	2.5	58	12.5%	0.20 [-0.88, 1.28]
Yu C et al., 2020	26.4	12	493	26.3	9.8	494	11.5%	0.10 [-1.27, 1.47]
Yudi MB et al., 2020	29.3	6	83	29.7	4.7	85	10.6%	-0.40 [-2.03, 1.23]
**Total (95% CI)**			**983**			**1003**	**100.0%**	**0.02 [-1.01, 1.05]**

Heterogeneity: Tau² = 1.92; Chi² = 64.88, df = 8 (P < 0.00001); I² = 88%
Test for overall effect: Z = 0.04 (P = 0.97)

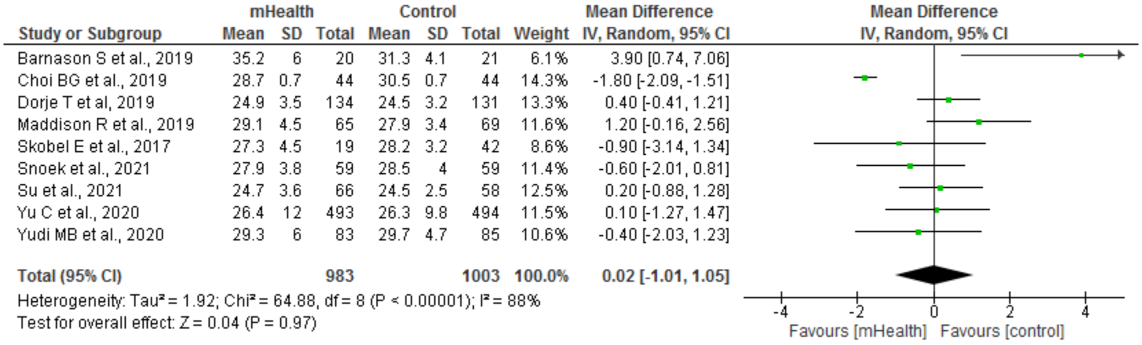

Waist circumference

Study or Subgroup	mHealth Mean	mHealth SD	mHealth Total	Control Mean	Control SD	Control Total	Weight	Mean Difference IV, Random, 95% CI
Maddison R et al., 2019	102.9	11.1	65	101.1	10.1	69	33.3%	1.80 [-1.80, 5.40]
Snoek et al., 2021	101	10	59	105	12	59	30.3%	-4.00 [-7.99, -0.01]
Su et al., 2021	89.67	9.43	58	91	8.29	58	36.4%	-1.33 [-4.56, 1.90]
**Total (95% CI)**			**182**			**186**	**100.0%**	**-1.10 [-4.22, 2.03]**

Heterogeneity: Tau² = 4.27; Chi² = 4.54, df = 2 (P = 0.10); I² = 56%
Test for overall effect: Z = 0.69 (P = 0.49)

Mean Difference
IV, Random, 95% CI

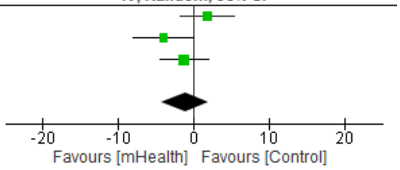

Supplement: Multimedia Appendix 6 [file mhealth_v10i12e39593_app6.pdf]
