# Supplementary material for: Effectiveness of mHealth Interventions in the Control of Lifestyle and Cardiovascular Risk Factors in Patients After a Coronary Event: Systematic Review and Meta-analysis
Source: JMIR Mhealth Uhealth. 2022 Dec 2;10(12):e39593. doi: 10.2196/39593 (PMC9758644; doi:10.2196/39593)

**Supplementary figures S4.** Forest plots for changes in HbA1c, glucose, heart rate and smoking cessation.

## HbA1c

| Study or Subgroup | mHealth Mean | mHealth SD | mHealth Total | Control Mean | Control SD | Control Total | Weight | Mean Difference IV, Random, 95% CI |
|---|---|---|---|---|---|---|---|---|
| Choi BG et al., 2019 | 5.8 | 0.1 | 44 | 6.1 | 0.1 | 44 | 44.4% | -0.30 [-0.34, -0.26] |
| Frederix I et al, 2017 | 5.8 | 0.7 | 62 | 6 | 1.1 | 64 | 26.1% | -0.20 [-0.52, 0.12] |
| Yudi MB et al., 2020 | 5.9 | 0.9 | 83 | 5.8 | 0.9 | 85 | 29.5% | 0.10 [-0.17, 0.37] |
| **Total (95% CI)** | | | **189** | | | **193** | **100.0%** | **-0.16 [-0.41, 0.10]** |

Heterogeneity: Tau² = 0.04; Chi² = 8.41, df = 2 (P = 0.01); I² = 76%
Test for overall effect: Z = 1.21 (P = 0.23)

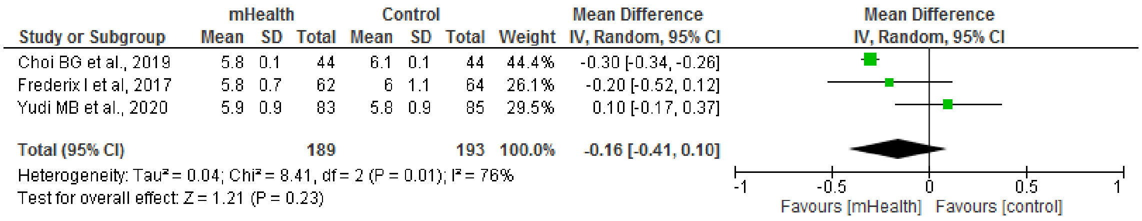


## Glucose

| Study or Subgroup | mHealth Mean | mHealth SD | mHealth Total | Control Mean | Control SD | Control Total | Weight | Mean Difference IV, Random, 95% CI |
|---|---|---|---|---|---|---|---|---|
| Frederix I et al, 2017 | 112 | 25 | 62 | 117 | 43 | 64 | 40.4% | -5.00 [-17.24, 7.24] |
| Maddison R et al., 2019 | 105.7 | 29.2 | 68 | 109.3 | 42.3 | 72 | 42.1% | -3.60 [-15.58, 8.38] |
| Skobel E et al., 2017 | 104.7 | 22.3 | 15 | 98.4 | 44 | 34 | 17.5% | 6.30 [-12.30, 24.90] |
| **Total (95% CI)** | | | **145** | | | **170** | **100.0%** | **-2.44 [-10.21, 5.34]** |

Heterogeneity: Tau² = 0.00; Chi² = 1.05, df = 2 (P = 0.59); I² = 0%
Test for overall effect: Z = 0.61 (P = 0.54)

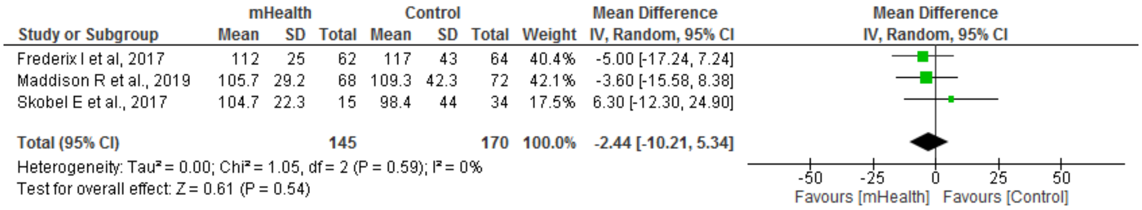


## Heart rate

| Study or Subgroup | mHealth Mean | mHealth SD | mHealth Total | Control Mean | Control SD | Control Total | Weight | Mean Difference IV, Random, 95% CI |
|---|---|---|---|---|---|---|---|---|
| Dorje T et al, 2019 | 68.9 | 8.2 | 134 | 74.4 | 10.3 | 131 | 42.9% | -5.50 [-7.74, -3.26] |
| Skobel E et al., 2017 | 64 | 10 | 19 | 64 | 10 | 42 | 22.9% | 0.00 [-5.42, 5.42] |
| Yudi MB et al., 2020 | 65.8 | 11.3 | 83 | 67.3 | 11.7 | 85 | 34.2% | -1.50 [-4.98, 1.98] |
| **Total (95% CI)** | | | **236** | | | **258** | **100.0%** | **-2.87 [-6.33, 0.59]** |

Heterogeneity: Tau² = 5.95; Chi² = 5.77, df = 2 (P = 0.06); I² = 65%
Test for overall effect: Z = 1.63 (P = 0.10)

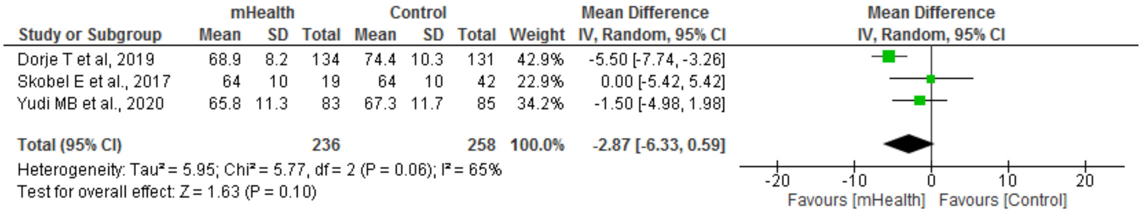


## Smoking cessation

| Study or Subgroup | mHealth Events | mHealth Total | Control Events | Control Total | Weight | Risk Difference IV, Fixed, 95% CI |
|---|---|---|---|---|---|---|
| Dorje T et al, 2019 | 102 | 116 | 120 | 131 | 12.9% | -0.04 [-0.11, 0.04] |
| Johnston N et al., 2016 | 75 | 81 | 65 | 72 | 9.3% | 0.02 [-0.07, 0.11] |
| Yu C et al., 2020 | 458 | 493 | 458 | 494 | 71.4% | 0.00 [-0.03, 0.03] |
| Yudi MB et al., 2020 | 71 | 83 | 72 | 85 | 6.4% | 0.01 [-0.10, 0.12] |
| **Total (95% CI)** | | **773** | | **782** | **100.0%** | **-0.00 [-0.03, 0.03]** |
| Total events | 706 | | 715 | | | |

Heterogeneity: Chi² = 1.19, df = 3 (P = 0.76); I² = 0%
Test for overall effect: Z = 0.05 (P = 0.96)

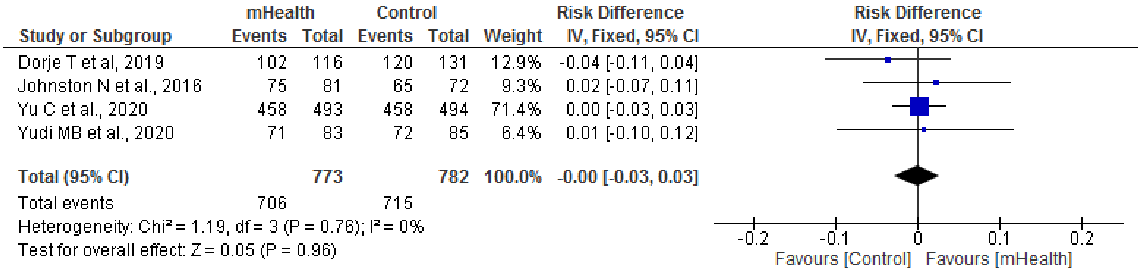

Supplement: Multimedia Appendix 7 [file mhealth_v10i12e39593_app7.pdf]
