# Supplementary figures and images for: Effectiveness of mHealth Interventions in the Control of Lifestyle and Cardiovascular Risk Factors in Patients After a Coronary Event: Systematic Review and Meta-analysis
Source: JMIR Mhealth Uhealth. 2022 Dec 2;10(12):e39593. doi: 10.2196/39593 (PMC9758644; doi:10.2196/39593)

Supplementary figures S5. Forest plots for changes in Vo2 peak.

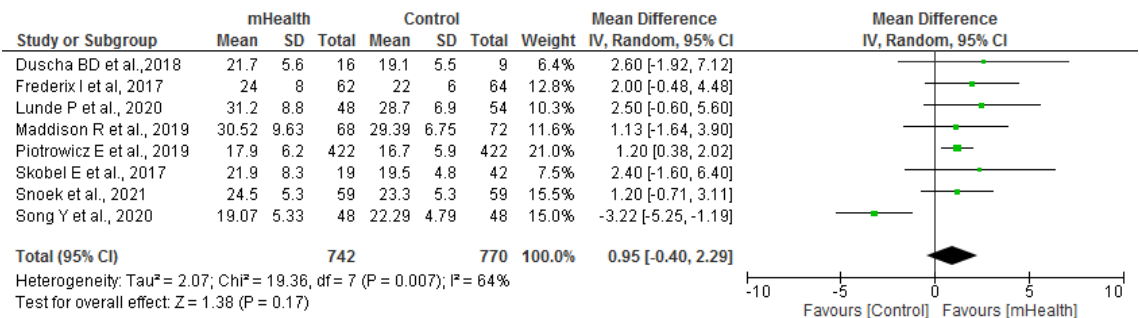

Supplement: Multimedia Appendix 8 [file mhealth_v10i12e39593_app8.pdf]

Supplementary figures S7. Forest plot for changes in mortality.

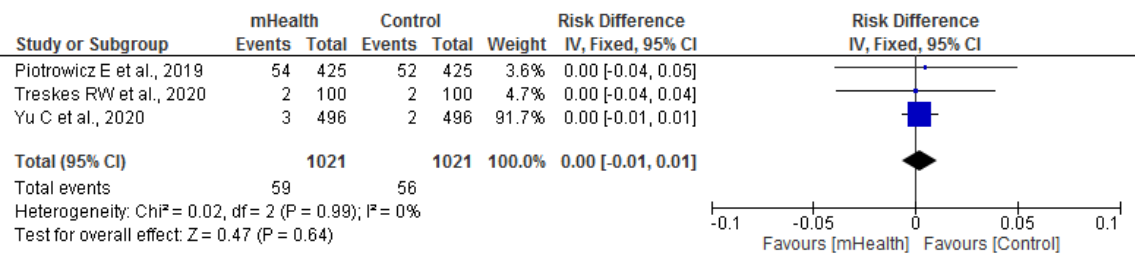

Supplement: Multimedia Appendix 10 [file mhealth_v10i12e39593_app10.pdf]
