# Supplementary material for: Effectiveness of mHealth Interventions in the Control of Lifestyle and Cardiovascular Risk Factors in Patients After a Coronary Event: Systematic Review and Meta-analysis
Source: JMIR Mhealth Uhealth. 2022 Dec 2;10(12):e39593. doi: 10.2196/39593 (PMC9758644; doi:10.2196/39593)

Supplementary figures S6. Forest plots for changes in anxiety, depression and quality of life.

Anxiety

Study or Subgroup	mHealth Mean	mHealth SD	mHealth Total	Control Mean	Control SD	Control Total	Weight	Std. Mean Difference IV, Random, 95% CI
Dorje T et al, 2019	2.5	3.1	134	2.8	3.2	131	44.0%	-0.09 [-0.34, 0.15]
Skobel E et al., 2017	19.3	1.9	19	19.2	3.2	42	8.7%	0.03 [-0.51, 0.58]
Snoek et al., 2021	3.2	3.2	59	4.2	3.9	59	19.4%	-0.28 [-0.64, 0.08]
Yudi MB et al., 2020	8.4	7.2	83	8.2	6.8	85	27.9%	0.03 [-0.27, 0.33]
**Total (95% CI)**			**295**			**317**	**100.0%**	**-0.08 [-0.24, 0.07]**

Heterogeneity: Tau² = 0.00; Chi² = 1.83, df = 3 (P = 0.61); I² = 0%
Test for overall effect: Z = 1.04 (P = 0.30)

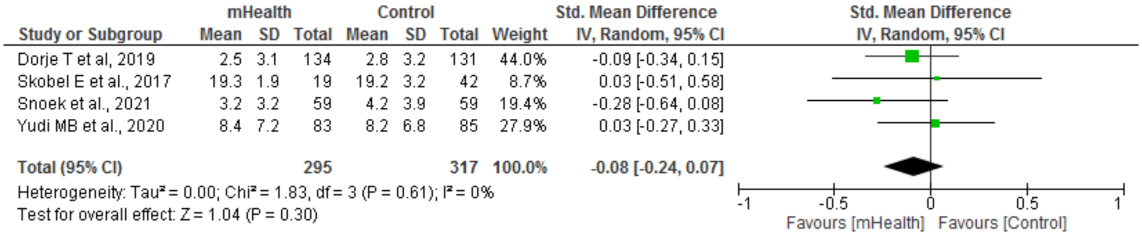

Depression

Study or Subgroup	mHealth Mean	mHealth SD	mHealth Total	Control Mean	Control SD	Control Total	Weight	Std. Mean Difference IV, Random, 95% CI
Dorje T et al, 2019	2.9	3	134	3.6	3.8	131	30.3%	-0.20 [-0.45, 0.04]
Fang J et al., 2018	57.8	17.4	33	50.6	26.7	34	13.6%	0.31 [-0.17, 0.80]
Skobel E et al., 2017	16.6	1.1	19	16.6	1.8	42	11.4%	0.00 [-0.54, 0.54]
Snoek et al., 2021	2.3	2.9	59	3	3.4	59	20.1%	-0.22 [-0.58, 0.14]
Yudi MB et al., 2020	68.5	25.1	83	64.2	25.4	85	24.5%	0.17 [-0.13, 0.47]
**Total (95% CI)**			**328**			**351**	**100.0%**	**-0.02 [-0.23, 0.19]**

Heterogeneity: Tau² = 0.02; Chi² = 6.66, df = 4 (P = 0.16); I² = 40%
Test for overall effect: Z = 0.20 (P = 0.84)

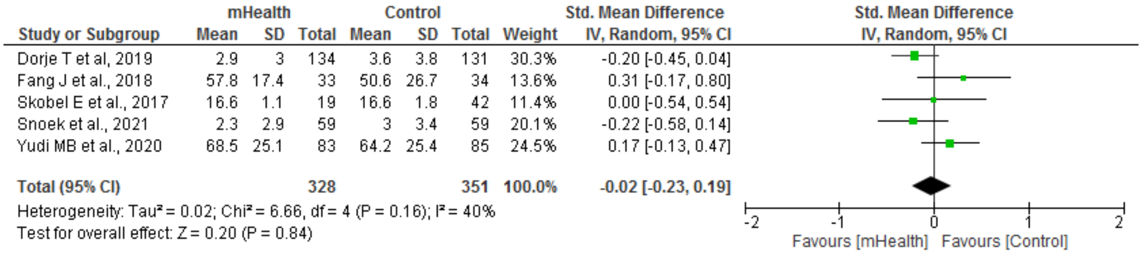

Quality of life. General

Study or Subgroup	mHealth Mean	mHealth SD	mHealth Total	Control Mean	Control SD	Control Total	Weight	Std. Mean Difference IV, Random, 95% CI
Hong et al., 2021	87.67	4.25	30	87.73	7.03	30	6.6%	-0.01 [-0.52, 0.50]
Johnston N et al., 2016	82.7	11.6	80	78.2	15.3	71	11.9%	0.33 [0.01, 0.65]
Lunde P et al., 2020	78	16	48	75	12	54	9.5%	0.21 [-0.18, 0.60]
Maddison R et al., 2019	0.89	0.13	65	0.92	0.09	69	11.2%	-0.27 [-0.61, 0.07]
Piotrowicz E et al., 2019	91.2	12.8	417	88.9	14.4	416	21.4%	0.17 [0.03, 0.30]
Skobel E et al., 2017	76.3	15	19	74.5	12.9	42	5.9%	0.13 [-0.41, 0.67]
Snoek et al., 2021	5.9	0.8	59	5.8	0.9	59	10.4%	0.12 [-0.24, 0.48]
Su et al., 2021	5.9	0.58	66	5.58	0.76	58	10.5%	0.47 [0.12, 0.83]
Yudi MB et al., 2020	77	14.4	83	79.8	16.1	85	12.6%	-0.18 [-0.49, 0.12]
**Total (95% CI)**			**867**			**884**	**100.0%**	**0.11 [-0.04, 0.26]**

Heterogeneity: Tau² = 0.02; Chi² = 15.20, df = 8 (P = 0.06); I² = 47%
Test for overall effect: Z = 1.46 (P = 0.15)

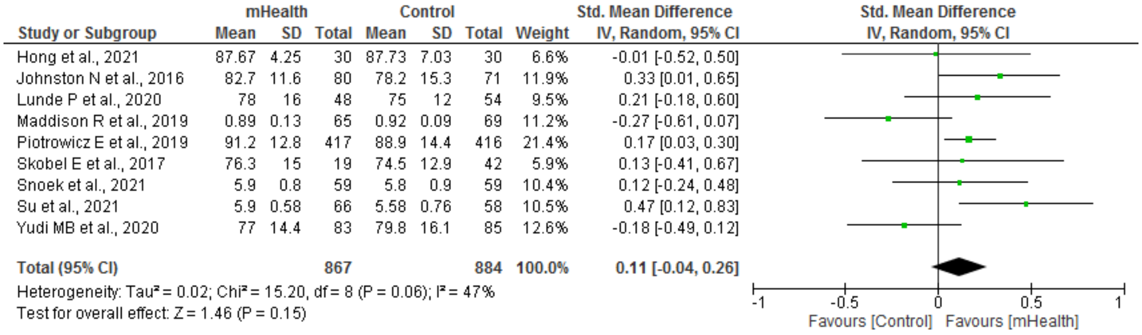

Supplement: Multimedia Appendix 9 [file mhealth_v10i12e39593_app9.pdf]
