# Supplementary material for: Effectiveness of mHealth Interventions in the Control of Lifestyle and Cardiovascular Risk Factors in Patients After a Coronary Event: Systematic Review and Meta-analysis
Source: JMIR Mhealth Uhealth. 2022 Dec 2;10(12):e39593. doi: 10.2196/39593 (PMC9758644; doi:10.2196/39593)
Supplement: Multimedia Appendix 11 [file mhealth_v10i12e39593_app11.pdf]

Supplementary Table S4. Sensitivity analysis.

6 minutes walk test sensitivity analysis

Study removed from meta-analysis	Mean difference	CI 95%	p
Dorje et al., 2019	21.76	11.30-32.23	0.0001
Fang et al., 2018	20.51	9.99-31.03	0.0001
Piotrowicz et al., 2019	23.76	12.54-34.97	0.0001
Yudi et al., 2020	20.96	11.69-30.23	0.00001

Quality of life. Physical dimension sensitivity analysis

Study removed from meta-analysis	Mean difference	CI 95%	p
Dorje et al., 2019	0.28	0.01-0.54	0.04
Fang et al., 2018	0.22	0.06-0.39	0.009
Frederix et al., 2017	0.22	0.01-0.44	0.04
Hong et al., 2021	0.30	0.13-0.47	0.0004
Lunde et al., 2020	0.29	0.07-0.51	0.009

Quality of life. Mental dimension sensitivity analysis

Study removed from meta-analysis	Mean difference	CI 95%	p
Dorje et al., 2019	0.32	0.04-0.60	0.03
Fang et al., 2018	0.19	0.02-0.35	0.03
Frederix et al., 2017	0.30	0.03-0.57	0.03
Hong et al., 2021	0.31	0.07-0.54	0.010
Lunde et al., 2020	0.27	0.00-0.53	0.05

Physical activity sensitivity analysis

Study removed from meta-analysis	Mean difference	CI 95%	p
Duscha et al., 2018	0.38	-0.07-0.84	0.09
Lunde et al., 2020	0.47	-0.11-1.05	0.11
Snoek et al., 2021	0.59	0.27-0.91	0.0003
Su et al., 2021	0.23	-0.08-0.54	0.14

Rehospitalization all causes sensitivity analysis

Study removed from meta-analysis	Mean difference	CI 95%	p
Piotrowicz et al., 2019	-0.03	-0.06-0.00	0.07
Widmer et al., 2017	-0.02	-0.05-0.00	0.07
Yu et al., 2020	-0.05	-0.10-0.01	0.10
Yudi et al., 2020	-0.03	-0.05-0.00	0.05

Rehospitalization cardiovascular causes sensitivity analysis

Study removed from meta-analysis	Mean difference	CI 95%	p
Piotrowicz et al., 2019	-0.03	-0.07-0.01	0.15
Snoek et al., 2021	-0.03	-0.07-0.00	0.06
Su et al., 2021	-0.05	-0.09 - -0.01	0.02
Treskes et al., 2020	-0.03	-0.07-0.00	0.08
Widmer et al., 2017	-0.03	-0.06 – 0.01	0.11
Yudi et al., 2020	-0.04	-0.07 - -0.00	0.05
